# MSDF-Net: a cross-version lightweight detection framework based on deformable convolution and high-resolution feature enhancement for pine wilt disease

**DOI:** 10.3389/fpls.2026.1857579

**Published:** 2026-06-18

**Authors:** Xiao Xiao, Yuxuan Lin, Simin Wang, Haifeng Lin, Fang Wang

**Affiliations:** 1 College of Information Science and Technology, Nanjing Forestry University, Nanjing, China; 2 College of Electronic Engineering, Nanjing Xiaozhuang University, Nanjing, China

**Keywords:** cross-version transferability, deformable convolution, lightweight, pine wilt disease, small target detection

## Abstract

**Introduction:**

Early and precise identification of pine wilt disease is critical for effective control. However, early-stage lesions are extremely small, sparse, and scattered, making them highly susceptible to being obscured by noise in complex forest backgrounds. Moreover, feature simplification in lightweight deployment further leads to the loss of critical pathological information.

**Methods:**

We propose MSDF-Net, a lightweight object detection framework that integrates a high-resolution P2 detection layer for enhanced small-target sensitivity, DCNv4-based deformable convolution for adaptive modeling of irregular spatial patterns, an EMA attention mechanism for background suppression, and a dual-branch C2f DualConv module for efficient multi-scale feature fusion. The model was evaluated on a cross-regional dataset spanning three provinces and two pine species.

**Results:**

MSDF-Net achieves an mAP@0.5 of 80.1%, outperforming the YOLOv8n baseline by 5.1 percentage points while maintaining 2.67M parameters and 11.7 GFLOPs. The most substantial improvement occurs in early-stage disease detection (PWD-E), with an AP gain of 20.2 percentage points. Cross-version validation on YOLOv11n, YOLOv12n, and YOLOv13n yields consistent improvements of 6.1, 4.3, and 5.1 percentage points in mAP@0.5, respectively.

**Discussion:**

Given its effectiveness across multiple YOLO versions and ecological conditions, MSDF-Net provides a generalizable solution with low parameter count and moderate computational complexity, making it a promising candidate for future UAV edge deployment pending on-device validation.

## 1 Introduction

Pine wilt disease (PWD), caused by the pine wood nematode, is one of the most destructive forest diseases worldwide, often termed the “cancer” of pine trees (Back et al., 2024). Characterized by high pathogenicity, rapid spread, and difficult control, this global epidemic frequently causes large-scale pine mortality within three to five years, resulting in severe economic and ecological losses (Zhou et al., 2022). Originating in North America, the pine wood nematode was first reported in Japan in 1905 and subsequently spread to East Asia and Europe via timber trade, becoming widespread in countries such as China, South Korea, and Portugal (Back et al., 2024; Li et al., 2022). In China, it was first detected in 1982 at the Sun Yat-sen Mausoleum in Nanjing and had spread to 19 provinces by 2022, causing massive tree mortality and posing serious threats to forest ecosystems, with average annual economic losses reaching 7.17 billion RMB between 1998 and 2017 (Zhou et al., 2022; Xu et al., 2023).

Traditional PWD monitoring relies on manual ground surveys, which are inefficient, costly, and limited in both coverage and timeliness (Zhou et al., 2022). Early-stage symptoms—such as slight needle chlorosis and wilting—are subtle and can easily be missed during field surveys, which makes large-scale early warning difficult in practice. Infected trees usually die within three months. Needles begin to yellow about three weeks after infection, then gradually turn reddish-brown while remaining attached to the branches (Back et al., 2024). Because untreated infections almost always result in tree death, the time window for effective detection is very limited (Zhou et al., 2022).

Recent advances in remote sensing and deep learning have made image-based monitoring a practical approach for PWD detection, covering both satellite-based observation and ground-level model-assisted analysis (Li et al., 2024a).

Satellite platforms cover large areas and provide frequent revisits along with rich spectral information. As a result, multispectral or hyperspectral imagery is often combined with vegetation indices to distinguish diseased trees from healthy canopies. Spatiotemporal approaches that integrate spectral, temporal, and spatial features from multi-year high-resolution imagery have been shown to improve user accuracy from 67.7% to 81.2% at the individual-tree level, while maintaining a producer accuracy of 84.7% (Zhang et al., 2021). Hyperspectral leaf reflectance analysis has identified vegetation indices such as VARI, VIgreen, and NWI as effective early detection indicators, with the GRSAl index demonstrating the best timeliness and stability, as infected trees show increased red and mid-infrared reflectance within two months (Kim et al., 2018). However, spatial resolution and revisit cycle limitations constrain satellite-based early detection.

In contrast, UAV remote sensing provides high spatial resolution, operational flexibility, and low cost, enabling high-quality imaging at stand and individual-tree levels and making it a key tool for fine-scale PWD monitoring (Sun et al., 2022). The combination of multispectral and hyperspectral UAV imagery with random forest models has achieved classification accuracies above 0.91 and enabled pre-symptomatic detection (Iordache et al., 2020). Optimal imaging resolutions of 10 cm, 8 cm, and 4 cm have been identified across different infection stages, yielding accuracies of 79.48%, 89.59%, and 99.28%, with GNDVI and REP serving as optimal indices for early and middle stages, respectively (Pan et al., 2023). High-resolution RGB imagery at 0.15 m has also been combined with vegetation index thresholding and morphological processing, achieving 90% accuracy in individual-tree extraction (Zhou et al., 2020). Despite these advantages, UAV-based monitoring still faces challenges including small target size, complex backgrounds, and operator dependence. To address these issues, CNN-based deep learning methods have been increasingly adopted, including two-stage detectors such as R-CNN and single-stage detectors such as YOLO. Spectral band selection via LDA combined with Mask R-CNN has achieved 83.51% overall accuracy with 74.89% early detection accuracy (Li et al., 2023), while a 3D-Res CNN approach has achieved 88.11% overall accuracy and 72.86% for early detection (Yu et al., 2021). However, two-stage methods face inherent limitations in forestry applications, including low inference efficiency, limited robustness to complex scenes, and high computational cost, which hinder lightweight deployment.

To overcome these limitations, recent studies have increasingly adopted YOLO-based single-stage detectors for PWD identification, seeking to balance small-target detection, robustness to complex backgrounds, and model lightweightness. Attention mechanisms have been widely incorporated to suppress background interference; for instance, the integration of SE (Hu et al., 2018) and CBAM (Woo et al., 2018) modules into YOLOv5s with SloU loss has achieved 90.1% precision and 88.3% recall (Dong et al., 2024). However, such methods primarily rely on channel and spatial attention for color and texture features, lacking the ability to model irregular disease shapes. Multi-strategy fusion approaches that integrate SE, CBAM, ECA (Wang et al., 2020), and SimAM (Yang et al., 2021) into YOLOv7, together with sliding window and dilated prediction, have achieved 92.81% precision and 89.58% recall. However, the associated computational cost remains high, which limits their practicality for lightweight deployment (Zhu et al., 2024). Researchers have also explored more lightweight designs by incorporating CBAM and CA (Hou et al., 2021) attention modules with BiFPN (Tan et al., 2020) and C2f-Faster-EMA in a PWD-YOLOv8n framework, achieving 94.3% mAP@0.5 (Su et al., 2024). Nevertheless, this design still relies on conventional attention mechanisms, and its effectiveness for early-stage small target detection is only partially verified. In addition, its robustness under complex background conditions has not been sufficiently evaluated.

From an architectural perspective, the YOLO series has undergone significant structural changes in different versions. YOLOv8 employs the C2f block, which is a cross-stage partially connected module consisting of two convolutions, as the core unit for feature extraction. This module segments the feature maps, processes them through a stacked bottleneck module based on standard 3 × 3 convolutions, and finally connects the results together. This modular layout enables the architecture to be modified in a targeted manner: the 3 × 3 convolution within each bottleneck can be replaced by other modules. For instance, deformable convolutions that can adaptively sample spatial positions can be used, and components such as attention mechanisms can also be inserted between functional modules without disrupting the entire process. YOLOv11, YOLOv12 and YOLOv13, as the newer members of this series, adopt the module based on C3k2. Their internal routing follows the pattern of Split → Bottleneck/C3k → Concat pattern, and each version gradually adds dedicated components to enrich the feature representation. The structural differences among these members highlight a practical requirement: The proposed detection improvement we have proposed not only needs to perform well on a single architecture, but also must have cross-version transferability across YOLO variants, rather than relying on a specific version of the backbone.

Apart from the considerations at the architectural level, due to the characteristics of forestry goals and their surrounding environment, the detection of PWD in drone images still faces significant challenges in essence. The main difficulties lie in the following two aspects:

1. Insufficient feature representation for small-scale and irregular targets
The early lesion areas are usually only a few pixels in size and have features such as blurred boundaries and irregular shapes. When the network extracts deep features, these weak signals are easily masked by the stronger background information around them, resulting in inaccurate positioning and missed detections.
2. Limited robustness and efficiency under complex backgrounds
The forest scene has diverse terrain and overlapping tree canopies. The similarity in appearance between diseased plants and healthy vegetation makes feature recognition more challenging. Additionally, the application based on drones requires lightweight models, so a balance must be struck between detection accuracy and onboard computing resources.

To address these challenges, a lightweight detection framework for pine wilt disease in UAV imagery is proposed, focusing on enhancing feature representation for small and irregular targets and improving robust, efficient modeling under complex backgrounds. The main contributions are as follows:

1. Feature enhancement for small-scale and irregular targetsTo enhance the ability to characterize early, small and irregular-shaped disease areas, we integrated DCNv4 into the high-level feature extraction stage, enabling the network to adaptively model geometric deformations. Additionally, we introduced a P2 detection layer, which utilizes high-resolution shallow features to enhance the perception of small targets and reduce missed detections.
2. Robust and lightweight feature modeling under complex backgroundsIn order to suppress background interference without sacrificing efficiency, we adopted the EMA attention mechanism, which highlights disease-related features and suppresses noise through cross-dimensional interaction. At the same time, we also used the C2f DualConv structure, which maintains strong feature representation capabilities while reducing computational redundancy, thereby achieving a better balance between detection accuracy and the efficiency required for drone deployment.

## 2 Materials and methods

### 2.1 Dataset description

**Figure 1:**
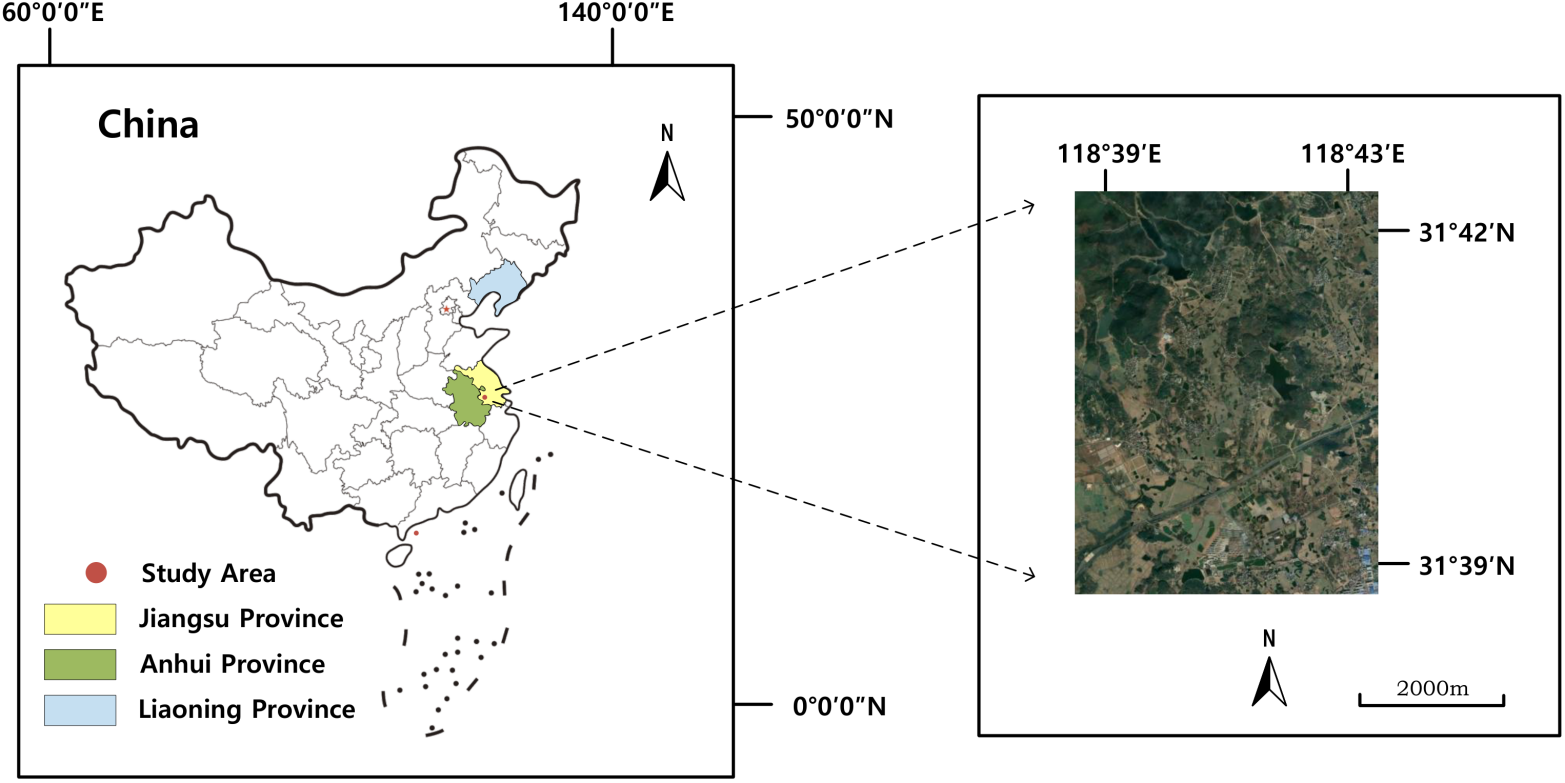
Geographic location of the study areas and UAV image sample. Data were collected across three provinces: Jiangsu, Anhui, and Liaoning, covering both subtropical and temperate climate zones with different dominant pine species.

The dataset of pine wood nematode disease images used in this study was collected from Jiangsu, Anhui and Liaoning provinces in China. The geographical area spans from north to south, covering significant differences in climate zones and pine forest types, as shown in **Figure 1**. The multi-region collection strategy effectively enhanced the ecological diversity and representativeness of the data set, providing a more reliable basis for evaluating the robustness of the model in different environments.

The southern study sites lie in Jiangsu Province and Anhui Province, both within the subtropical monsoon climate zone where warm, humid conditions favor the survival and spread of the pine wood nematode (*Bursaphelenchus xylophilus*). *Pinus massoniana* (Masson pine) dominates these regions and is the most widely distributed pine species PWD affects in southern China. Masson pine forests here grow under dense canopy cover mixed with broadleaf species, a combination that introduces considerable visual heterogeneity and background interference into UAV imagery.

The northern study site is located in Liaoning Province, a temperate monsoon zone where winters are colder and seasonal contrasts are sharp. The dominant tree species in this area is *Pinus thunbergii* (Japanese black pine), and its needle shape, canopy structure and disease development process are all different from those of *Pinus massoniana*. Trees of *Pinus thunbergii* infected with PWD often exhibit different coloration patterns and needle wilting rates from those of *Pinus massoniana*, which adds valuable interspecific difference information to the dataset. Therefore, incorporating data from Liaoning helps to verify the adaptability of this model when dealing with different ecological regions and host tree species.

During the data collection stage, we used a UAV (DJI Air 2) to capture RGB images from various flight heights and perspectives to ensure the diversity of the data in terms of scale and viewpoint. These pictures cover the entire course of PWD from the initial infection to the severe wilting stage, and include the forest scene under the conditions of stand density, topographical features and light environment. To facilitate the analysis, we have classified the disease into four stages based on the progression of the visual symptoms. PWD-E corresponds to the initial stage of infection, characterized by slight chlorosis of the needles, which is almost indistinguishable from that of healthy plants and belongs to the most difficult category to detect. PWD-M represents the transitional stage. The needle coloration becomes more distinct, presenting a mixture of green, yellow and brown tones. The tree crown structure begins to show a significant degree of thinning. PWD-L, at the advanced stage, the tree canopy shows a large area of reddish-brown color, the needles are severely withered, and the structure is significantly deteriorated. Even in medium-resolution images, it can be clearly identified. PWD-D is the final stage, where the trees have completely died, leaving only grayish-white dead branches or a small amount of brown, dry needles. These four stages cover the entire course of the disease, allowing us to assess how performance responds dynamically to changes in visual characteristics across each stage.

All UAV RGB images were manually annotated using the LabelImg tool to produce YOLO-format bounding boxes with stage-specific labels. The annotation team consisted of forestry graduate students supervised by forestry faculty, all of whom had prior field experience in pine wilt disease diagnosis and were trained on a shared annotation guideline before labeling began. Rather than performing independent parallel annotation, we adopted a consensus-based protocol designed to control labeling noise at its source. Under this protocol, every image was first annotated by one annotator, and any sample flagged as ambiguous—most often early-stage cases where the boundary between slight chlorosis and healthy foliage is inherently subtle—was then reviewed jointly by at least two additional annotators together with the supervising faculty. When the reviewers disagreed on stage classification or bounding box placement, the case was discussed until a single consensus label was reached. Samples for which no consensus could be achieved were excluded from the dataset to avoid contaminating training and evaluation with disputed labels. Because each final label represents an agreed decision rather than a single annotator’s opinion, conventional inter-annotator agreement statistics such as Cohen’s kappa or pairwise IoU consistency are not directly applicable to the released annotations. The consensus protocol effectively shifts disagreement resolution to the labeling stage itself, which is particularly important for the PWD-E category where visual ambiguity is highest and where the reported AP improvements would otherwise be most sensitive to label noise.

**Figure 2:**
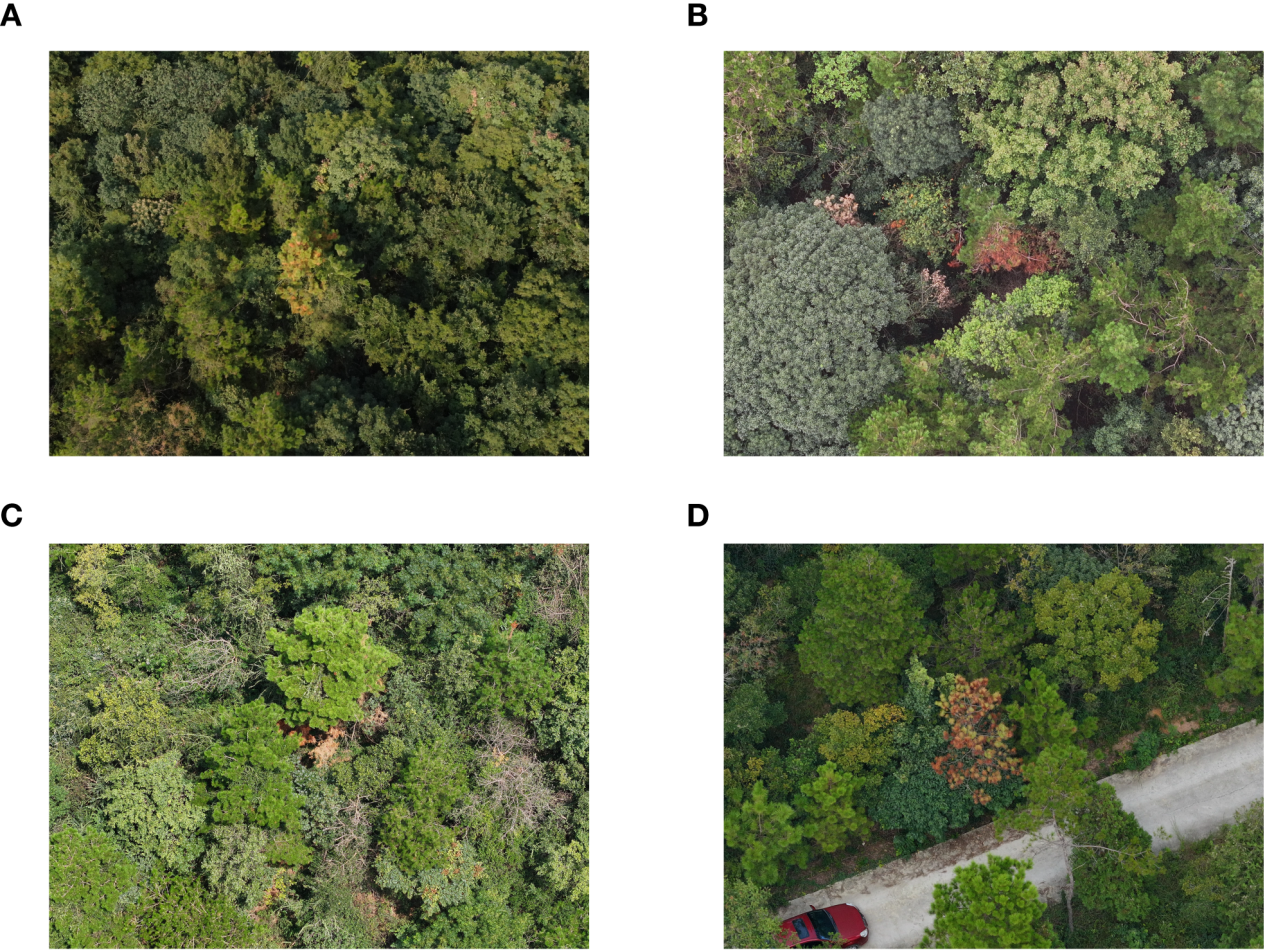
Representative detection challenges in UAV-based PWD monitoring. **(a)** Small early-stage target with subtle discoloration embedded in dense canopy; **(b)** irregularly shaped diseased region with blurred boundaries caused by canopy overlap; **(c)** dense canopy occlusion reducing the visibility of diseased trees; **(d)** background interference from man-made structures such as roads and vehicles.

When these four types of samples are examined in the context of a real forest environment, their imaging process encounters various dimensional disturbances and challenges. As illustrated in **Figure 2**, the main detection challenges in UAV forestry monitoring are as follows: The early-stage targets are small in size and the color changes are not obvious, making them difficult to identify in the dense tree canopy; the irregular shapes and blurred boundaries of the diseased areas caused by tree crown overlap; severe canopy obstruction resulting in only partial visible infected trees and background interference caused by artificial objects such as roads and vehicles. These varied and challenging conditions make the dataset a rigorous testbed for evaluating how robust and generalizable a detection model truly is. To ensure a fair and unbiased evaluation, careful attention was paid to how the dataset was partitioned, as described below.

The 1145 images were randomly partitioned into training, validation, and test sets, with 893 images (approximately 78%) allocated to the training set, 128 images (approximately 11%) to the validation set, and 124 images (approximately 11%) to the test set, corresponding to an approximate 8:1:1 ratio. The validation set was used for hyperparameter tuning and model selection, while the test set was held out exclusively for final evaluation and was never used during training.

Given the uneven geographic and categorical distribution of the data, which spans three provinces (Jiangsu, Anhui, Liaoning) and four disease stages (PWD-E, PWD-M, PWD-L, PWD-D), an unstratified random split could introduce systematic bias where certain disease stages or regions might be over- or under-represented in any given subset. To mitigate this risk, we employed stratified sampling across two dimensions simultaneously: disease stage and geographic province. For each unique combination of stage and province, samples were randomly assigned to training, validation, and test subsets while preserving the approximate 8:1:1 ratio. This ensured that each subset reflected the same stage–province distribution as the full dataset, preventing any disease stage or geographic region from being disproportionately represented in any split.

Pine species was not used as an explicit stratification variable because species distribution is largely confounded with province in our dataset: the southern provinces (Jiangsu and Anhui) are dominated by Masson pine (*Pinus massoniana*), while the northern province (Liaoning) is dominated by Japanese black pine (*Pinus thunbergii*). Stratifying by province therefore implicitly accounts for the major species-level variation, ensuring that both pine species are proportionally represented across all subsets.

The full dataset contains 1145 UAV images with 4894 annotated bounding boxes distributed across the four disease stages. The images were captured by a DJI Air 2 UAV at varying flight altitudes, yielding original resolutions ranging from approximately 4000 × 3000 to 5280 × 3956 pixels, all of which were resized to 640 × 640 pixels for model training and inference. The per-split image counts and per-stage bounding-box distribution are summarized in **Table 1**.

**Table 1:** Dataset composition: per-split image counts and per-stage bounding-box distribution.

Split	Images	PWD-E	PWD-M	PWD-L	PWD-D	Total boxes
Train	893	1805 (47.6%)	462 (12.2%)	888 (23.4%)	635 (16.8%)	3790
Val	128	280 (50.5%)	53 (9.6%)	143 (25.8%)	78 (14.1%)	554
Test	124	282 (51.3%)	53 (9.6%)	135 (24.5%)	80 (14.5%)	550
Total	**1145**	**2367 (48.4%)**	**568 (11.6%)**	**1166 (23.8%)**	**793 (16.2%)**	**4894**

Percentages within each row are relative to the row’s total bounding-box count. Bold values indicate the total counts across all dataset splits.

The dataset exhibits a natural class imbalance that reflects real-world PWD monitoring conditions. PWD-E (early-stage) samples are the most numerous, accounting for approximately 48% of all annotations, whereas PWD-M (mid-stage) samples are the least frequent at approximately 12%. This distribution is ecologically representative: early-stage infections, although subtle and difficult to detect, are widespread in affected forests, whereas mid-stage symptoms represent a relatively brief transitional phase that is less often captured during periodic UAV surveys. The stratified sampling strategy described above preserves this stage distribution across the training, validation, and test subsets, so the imbalance is reflected consistently in evaluation rather than concentrated in any single split.

The bounding-box size distribution, expressed as normalized box area relative to the 640 × 640 input dimensions, exhibits a substantial multi-scale gap between the disease stages. PWD-E targets are predominantly small, with a median normalized area of approximately 1.0 × 10^−4^ (corresponding to roughly 6 × 6 pixels at the 640 × 640 input resolution). In contrast, PWD-M, PWD-L, and PWD-D targets span a much wider size range, with median normalized areas approximately two orders of magnitude larger (around 0.02–0.03, corresponding to roughly 90 × 90 pixels). The smallest annotated PWD-E box occupies only 1.1 × 10^−5^ of the image area, confirming the extreme small-target challenge that motivates the introduction of the P2 high-resolution detection layer in this work.

### 2.2 Network architecture design

#### 2.2.1 Design motivation

Recent one-stage object detection frameworks have delivered notable gains in efficiency and usability (Hermens, 2024). But their performance remains limited in UAV-based pine wilt disease detection, where targets are small, disease morphology is irregular, and forest backgrounds are visually complex (Wang et al., 2023). A network that handles these conditions must combine strong small-target representation, resilience to background interference, and low computational cost.

To meet these requirements, we design a detection architecture that follows the standard one-stage layout: a feature extraction stage, a multi-scale feature fusion stage, and a detection head. Combining deep feature extraction with multi-scale aggregation in this way gives the model a solid foundation for efficient detection.

Applying such an architecture directly to pine wilt disease detection is not sufficient, because the network struggles to model irregular disease patterns, capture small-scale features, and filter out background noise. We therefore introduce targeted modifications tailored to the specific characteristics of this task.

#### 2.2.2 Overall architecture of MSDF-Net

**Figure 3:**
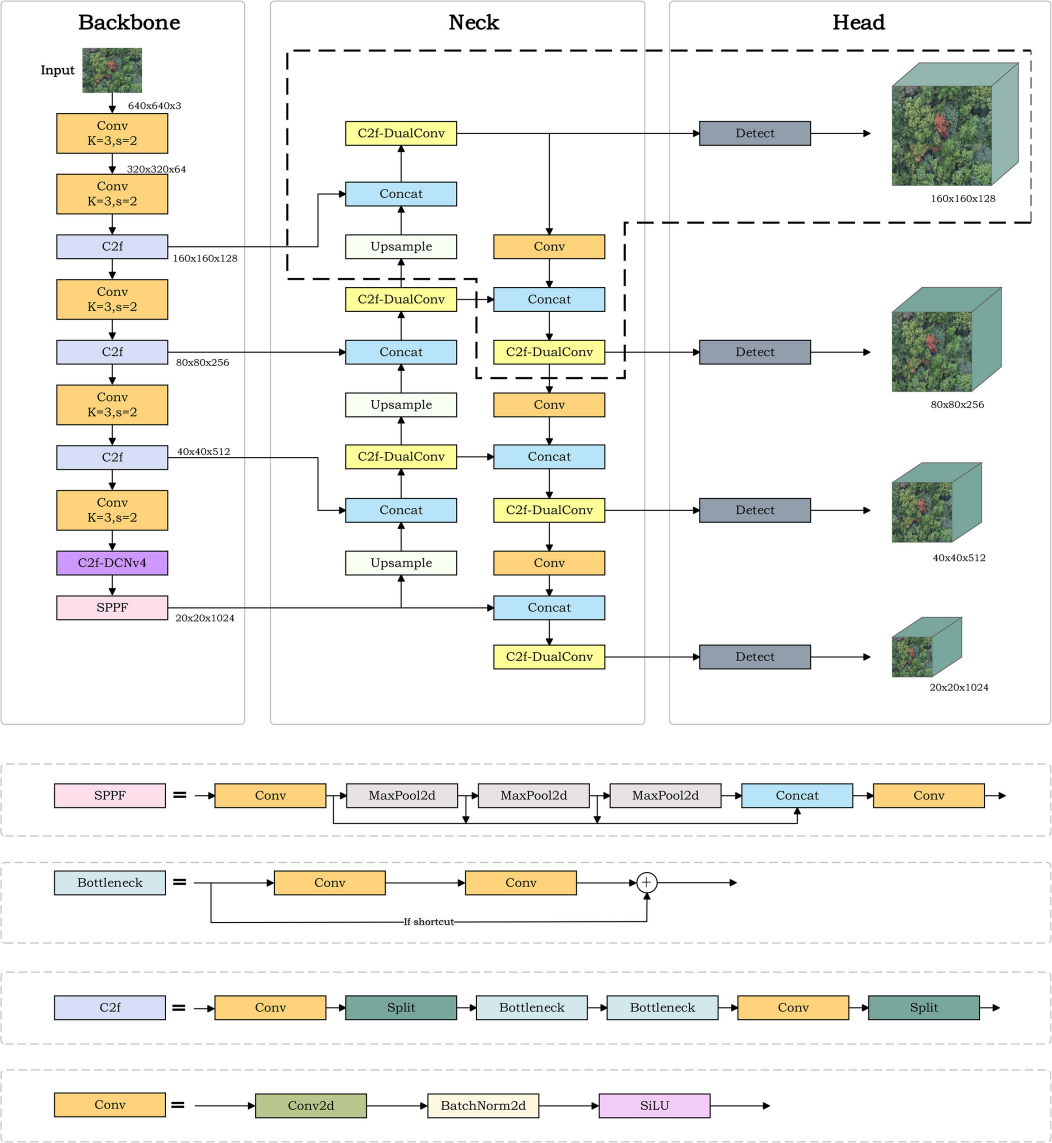
Overall architecture of the proposed MSDF-Net.

Building on these design considerations, we construct a lightweight detection network named MSDF-Net, as shown in **Figure 3**. The architecture targets two goals: strengthening feature representation for targets that are small or irregularly shaped, and improving both robustness and efficiency when the model operates against complex forest backgrounds.

To address the issue of insufficient representation of small-sized and irregular targets, we have jointly improved the feature extraction and detection scales at both levels. In the feature extraction stage, we integrate C2f_DCNv4 into the high-level network to achieve adaptive spatial sampling, enabling the model to better capture the geometric deformation and irregular boundaries of the diseased areas. Meanwhile, a P2 detection layer is introduced at the head of the detection process. By utilizing shallow features that contain more detailed spatial information, the model’s sensitivity to small-scale lesion areas is enhanced, and the omission of early targets is reduced.

For feature aggregation in complex backgrounds, we have designed a lightweight multi-scale fusion structure to reduce computational redundancy while maintaining efficiency. The C2f_DualConv module enhances feature interaction through dual convolution branches, enabling the network to integrate local details with global context information. Furthermore, at the end of the feature extraction stage, the EMA attention mechanism is introduced. By recalibrating the feature responses in both spatial and channel dimensions, it effectively highlights disease-related information and suppresses background noise.

The combined effect of these modules enables MSDF-Net to jointly optimize feature extraction, feature fusion, and multi-scale representation, achieving a good balance between detection accuracy and computational cost.

### 2.3 Feature enhancement for small-scale and irregular targets

Small and irregular disease targets in UAV imagery are prone to feature loss and weak representation, which often causes them to be missed. We address this from two aspects: adaptive modeling at higher-level stages and improved use of high-resolution features.

#### 2.3.1 Deformable convolution-based adaptive feature extraction

In UAV-based pine wilt disease detection, diseased regions often take on irregular geometric shapes because imaging angles vary, trees grow in different postures, and branches occlude parts of the crown (Yang et al., 2025). Standard convolutions sample on a fixed grid, so they struggle to capture the spatial variability of these disease patterns. This issue is especially evident for early-stage PWD targets, where boundaries are blurred and shapes are highly irregular. Enhancing the spatial adaptability of feature extraction is therefore essential for accurately modeling such targets (Kee et al., 2023).

In the standard YOLOv8 architecture, the C2f block serves as the core feature extraction unit. Given an input feature map *X*, the standard C2f block first applies a 1 × 1 convolution to reduce the channel dimension, splits the result into two branches, processes one branch through *N* sequential Bottleneck modules with standard 3 × 3 convolutions, and concatenates all outputs:

(1) $$F_{\text{C}2\text{f}}\left(X\right)={\text{Conv}}_{1\times 1}\left(\text{Concat}\left(X_{1},B_{1}\left(X_{2}\right),B_{2}\left(B_{1}\left(X_{2}\right)\right),\ldots ,B_{N}\left(\ldots \right)\right)\right)$$

where *X*_1_ and *X*_2_ are the two split branches, and *B_i_* denotes the *i*-th standard Bottleneck module using fixed-grid 3 × 3 convolutions. In this formulation, every Bottleneck samples features at predetermined grid positions, regardless of the actual spatial distribution of the target.

To adapt this structure for irregular PWD target modeling, the standard 3 × 3 convolutions within the Bottleneck modules are replaced with deformable convolutions, constructing the C2f_DCNv4 module. The modified Bottleneck operation is defined as Equation 2 and Equation 3:

(2) $$B_{i}^{\text{dcn}}\left(F\right)=Conv_{1\times 1}\left(,\text{ DCN}\left(Conv_{1\times 1}\left(F\right)\right)\right)$$

where DCN denotes the deformable convolution operation with learnable spatial offsets and modulation scalars:

(3) $$\text{DCN}\left(F,p_{0}\right)=\sum_{k=1}^{K}w_{k}\cdot F\left(p_{0}+p_{k}+\text{Δ}p_{k}\right)\cdot \text{Δ}m_{k}$$

Here, *p*_0_ is the current output position, *p_k_* represents the predefined sampling offset, Δ*p_k_* is the learnable spatial offset, and Δ*m_k_* is the modulation scalar controlling the contribution of each sampling point. Unlike the original DCNv4 formulation applied in general-purpose feature extraction (Xiong et al., 2024), in this work the deformable operation is specifically embedded within the C2f Bottleneck structure to serve as a targeted replacement for fixed-grid convolutions. The overall C2f DCNv4 module is thus formulated as:

(4) $$F_{\text{C}2\text{f}\_\text{DCNv}4}\left(X\right)={\text{Conv}}_{1\times 1}\left(\text{Concat}\left(X_{1},B_{1}^{\text{dcn}}\left(X_{2}\right),B_{2}^{\text{dcn}}\left(B_{1}^{\text{dcn}}\left(X_{2}\right)\right),\ldots ,B_{N}^{\text{dcn}}\left(\ldots \right)\right)\right)$$

Comparing Equation 4 with Equation 1, the key difference lies in the replacement of the fixed-grid Bottleneck *B_i_* with the deformable variant *B_i_*^dcn^. With this substitution, each Bottleneck dynamically adjusts its sampling positions to match the spatial distribution of input features, so the receptive field conforms to the irregular boundaries of diseased regions instead of relying on predetermined grid locations.

The C2f_DCNv4 module is placed at the 8th layer of the backbone, corresponding to the deepest feature extraction stage. This placement is motivated by two considerations specific to PWD detection. First, high-level feature maps encode abstract semantic information that provides a more reliable basis for offset learning—at shallow layers, features are dominated by low-level textures such as leaf edges and branch patterns that are shared between healthy and diseased trees, making offset estimation unreliable. Second, placing deformable convolution only at the deepest layer limits the computational overhead to a single stage, preserving the lightweight characteristics desirable for resource-constrained platforms while concentrating the adaptive sampling capability where it is most beneficial for distinguishing irregular disease patterns from complex backgrounds.

**Figure 4:**
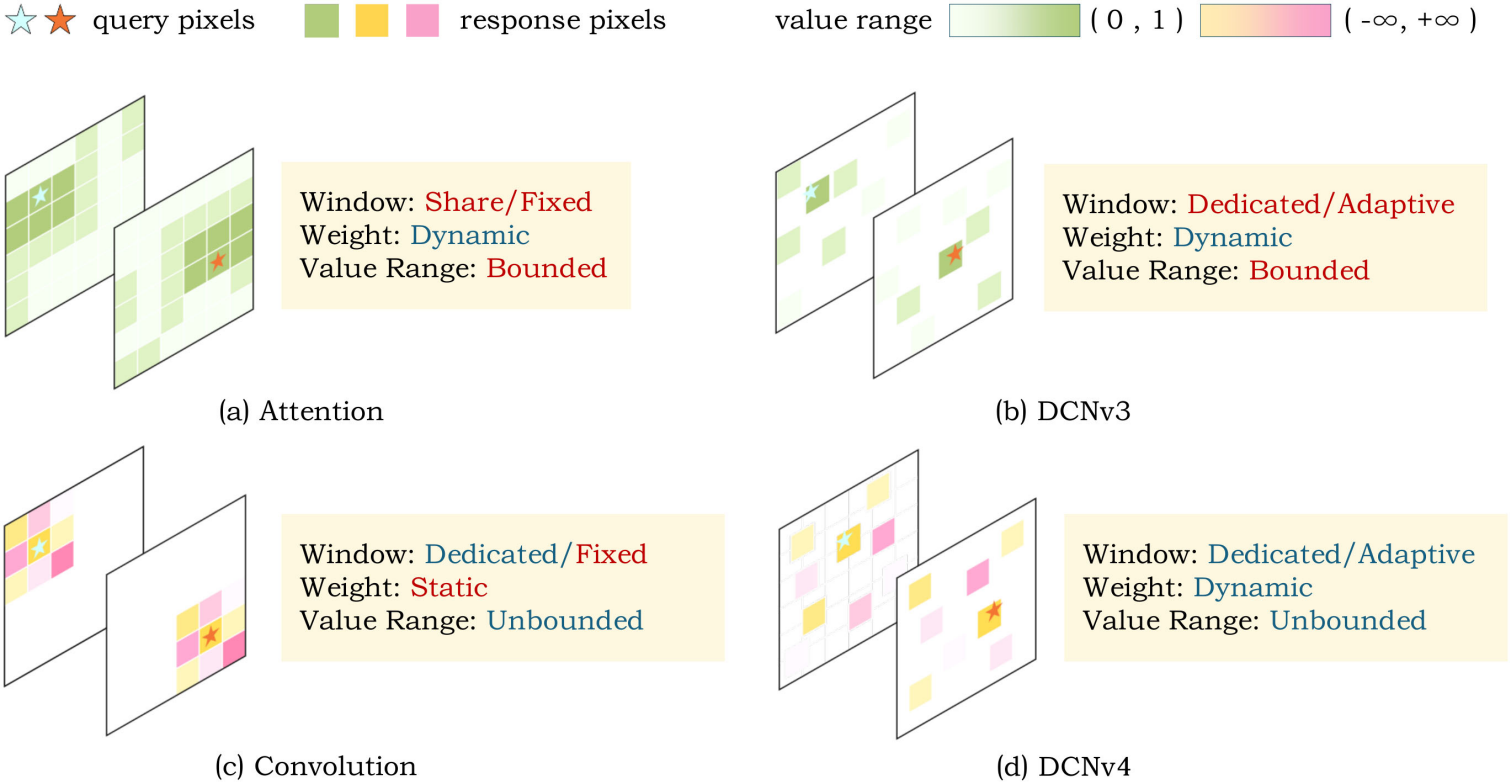
Comparison between DCNv4 and other spatial aggregation core operators.

Compared with standard convolution and attention mechanisms, the deformable convolution within C2f_DCNv4 offers a distinct advantage in spatial feature aggregation. Standard convolution lacks adaptability to geometric variations, while attention mechanisms model global dependencies at the cost of increased computational overhead. The deformable approach introduces spatial adaptability while maintaining local feature aggregation efficiency, making it particularly suited for the irregular and small-scale disease targets encountered in forestry monitoring. The differences among these mechanisms are illustrated in **Figure 4**.

**Figure 5:**
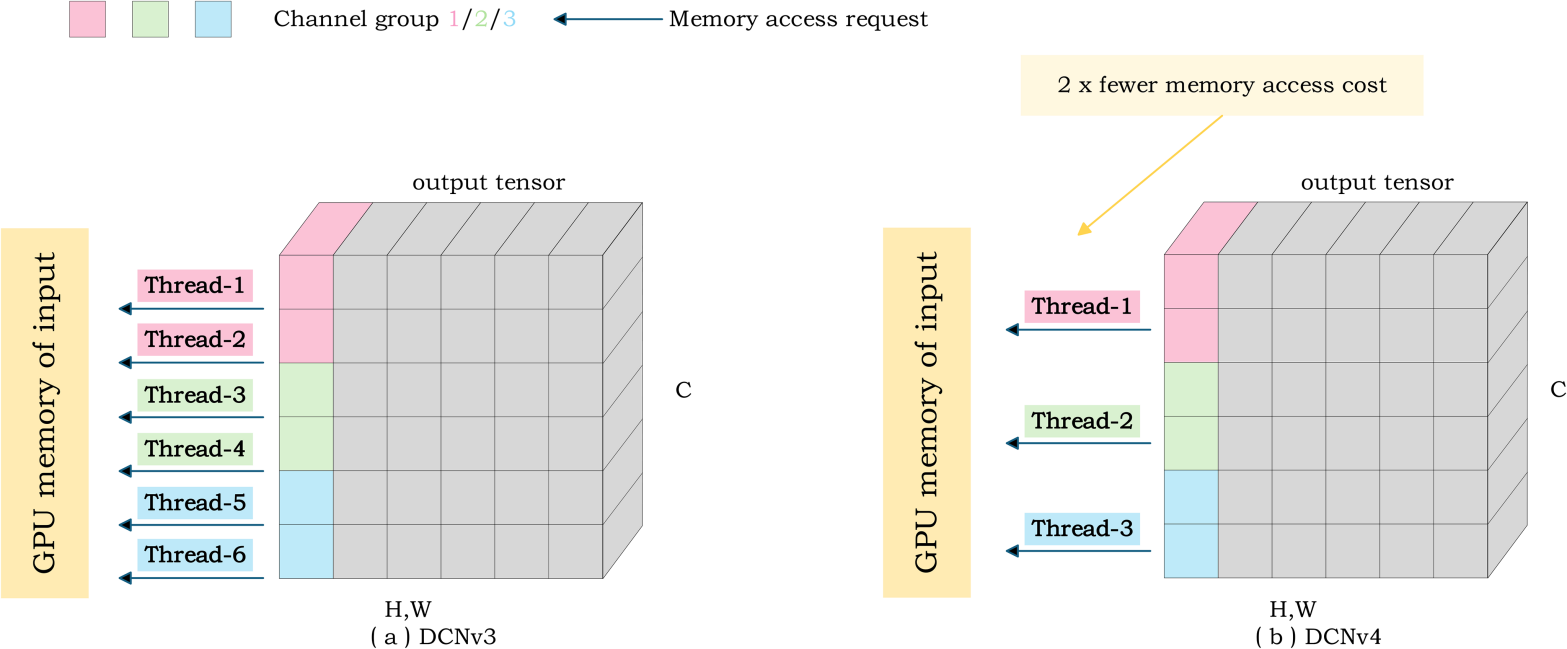
Improvement of DCNv4 in optimizing memory access. *H* stands for Height, *W* is Width, and C represents the number of Channels.

In terms of computational efficiency, the deformable convolution implementation follows an optimized aggregation strategy (Xiong et al., 2024) that reduces redundant memory access and improves parallel computation efficiency, effectively controlling computational cost while maintaining strong feature representation capability. The corresponding structural optimization is illustrated in **Figure 5**.

**Figure 6:**
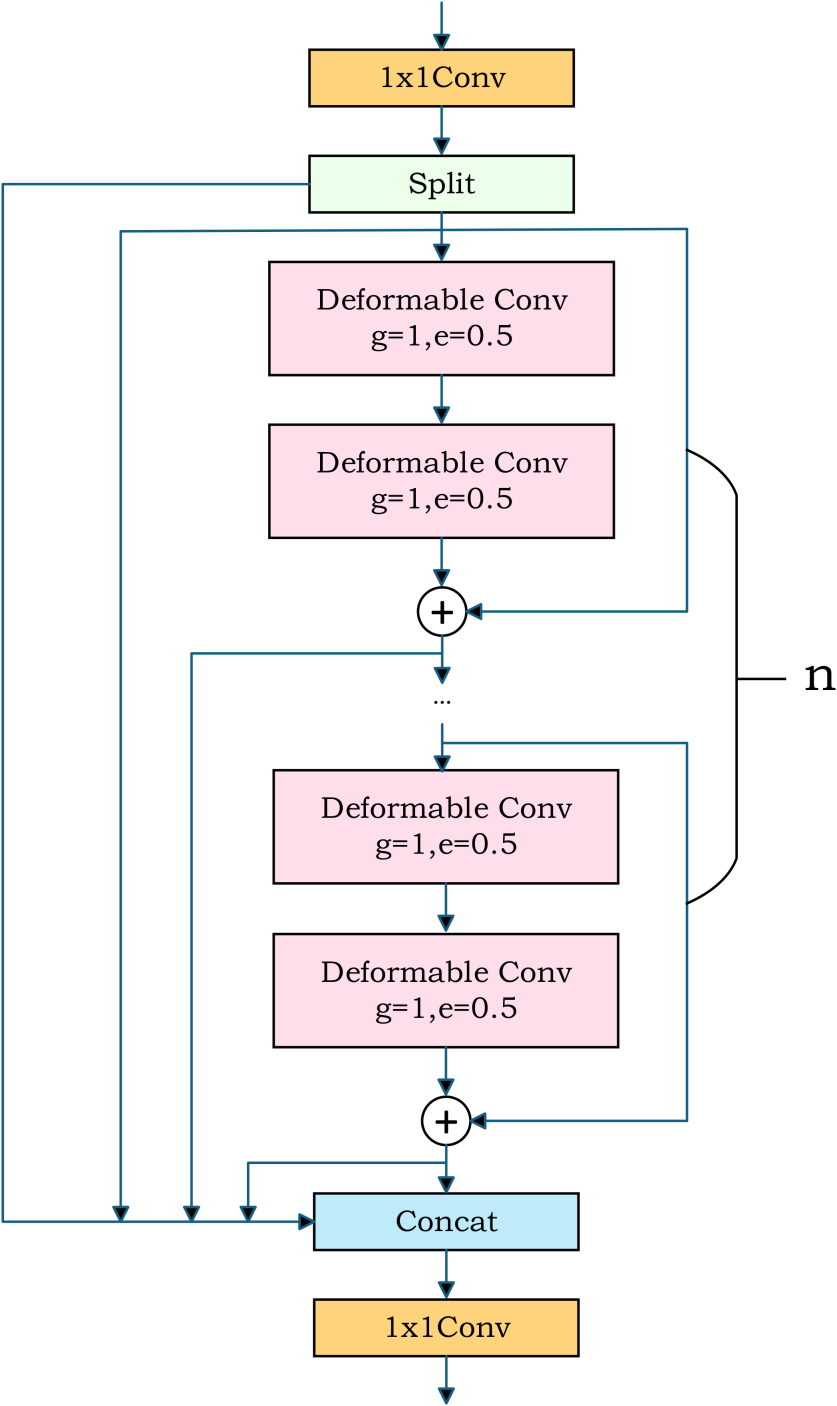
Structure of the proposed C2f DCNv4 module.

Based on the above design, **Figure 6** illustrates the overall structure of the C2f_DCNv4 module, where the deformable Bottleneck units replace the standard fixed-grid convolutions within the C2f framework, enabling adaptive spatial sampling for irregular PWD target feature extraction while preserving the efficient split-concatenate architecture of the original block.

#### 2.3.2 High-resolution feature enhancement via P2 detection layer

While adaptive feature extraction improves the modeling of irregular disease patterns, the representation of extremely small targets remains limited due to insufficient spatial resolution. Progressive downsampling in deep networks reduces fine-grained details, making small-scale disease regions difficult to localize accurately.

The original detection architecture of YOLOv8 typically adopts a three-scale detection strategy, performing object prediction on three feature maps—P3, P4, and P5—following the multi-scale feature pyramid structure (Lin et al., 2017). Although this structure can balance the detection requirements of objects at different scales to a certain extent, it exhibits limitations when handling smaller targets. As the network undergoes progressive downsampling, the spatial resolution of feature maps continuously decreases, and the information of small objects in high-level feature maps is prone to being compressed or lost, making it difficult for the model to accurately localize object boundaries.

Assuming the input image has size *H* × *W*, after a convolution operation with stride *s* the resulting feature map has size *H/s* × *W/s*. The YOLOv8 backbone performs four stages of downsampling with strides of 4, 8, 16, and 32, producing feature maps that correspond to P2 (*H/*4 × *W/*4), P3 (*H/*8 × *W/*8), P4 (*H/*16 × *W/*16), and P5 (*H/*32 × *W/*32), respectively. However, the original detection head only utilizes P3, P4, and P5 for object prediction, while the highest-resolution P2 layer is discarded. For pine wilt diseased trees, which are extremely small in UAV remote sensing images and easily submerged in complex backgrounds, deep convolutional operations significantly degrade spatial details. In particular, each pixel in the P5 layer corresponds to a 32 × 32 region in the original image, causing near-complete loss of fine-grained information for small targets and resulting in a high rate of missed detections.

Recent studies have attempted to enhance small-object perception by introducing high-resolution detection layers. A P2 detection layer has been introduced in YOLO11s alongside the removal of the redundant P5 layer, achieving a 3.4% improvement in mAP@0.5 and a 24.7% reduction in parameters, with the effectiveness of the P2 layer further validated on a tea bud small-object dataset when combined with the CSA attention mechanism (Wang et al., 2025). In a separate study, a P2 small-object detection layer based on shallow high-resolution features from the backbone network was incorporated with the ASF mechanism, improving mAP@0.5 by 0.036 and mAP@0.5:0.95 by 0.040, and when combined with Soft-NMS, significantly enhancing both detection accuracy and visual quality for small objects while maintaining parameters at 22.6M (Wang and Zhao, 2025).

Building on these findings, this work introduces a P2 small-object detection layer into the original three-scale detection head of YOLOv8, leveraging shallow high-resolution spatial representations to improve the detection capability for early-stage pine wilt diseased trees. The P2 layer is located after the first downsampling stage of the backbone network, with a feature map size of 160 × 160 for an input of 640 × 640, corresponding to a downsampling factor of 4. Theoretically, this enables the detection of extremely small targets with sizes larger than 4 × 4 pixels. By incorporating the Feature Pyramid Network (FPN) and Path Aggregation Network (PAN), the P2 layer is bidirectionally fused with the P3, P4, and P5 layers, enabling sufficient interaction of fine-grained spatial cues and high-level semantic context.

Let the feature map at layer *l* be denoted as *F_l_*. The top-down fusion process of FPN can be expressed as Equation 5:

(5) $$F_{l}^{\text{top}-\text{down}}=\text{Conv}\left(\text{Upsample}\left(F_{l+1}\right)⊕F_{l}\right)$$

where ⊕ denotes element-wise addition and Upsample represents the upsampling operation. The bottom-up fusion process of PAN further enhances localization information and can be expressed as Equation 6:

(6) $$F_{l}^{\text{bottom}-\text{up}}=\text{Conv}\left(\text{Downsample}\left(F_{l-1}^{\text{top}-\text{down}}\right)⊕F_{l}^{\text{top}-\text{down}}\right)$$

**Figure 7:**
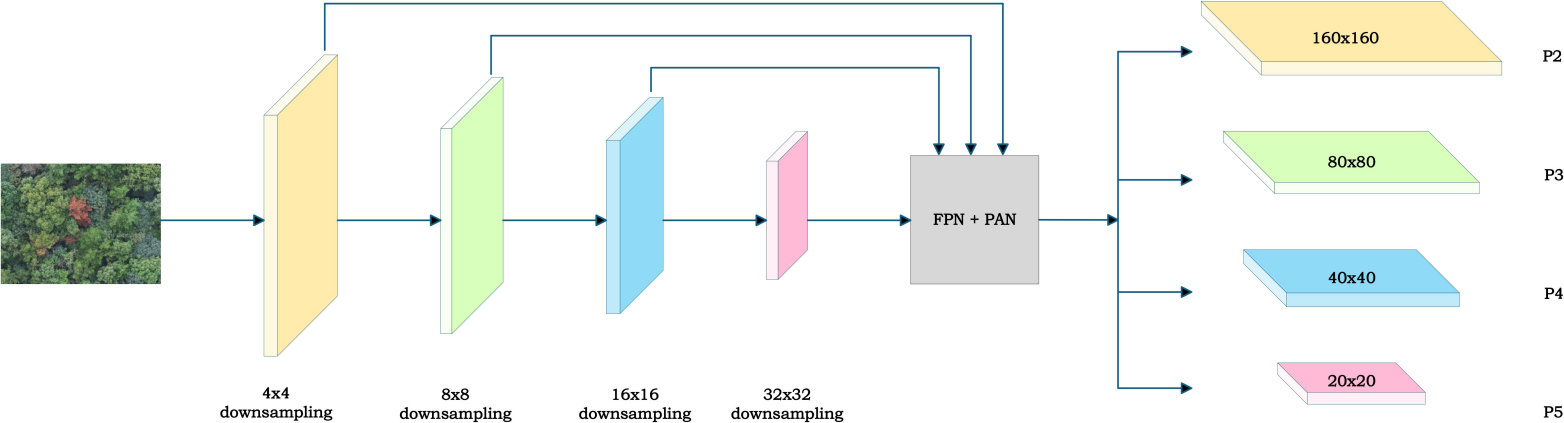
Feature extraction network with P2 detection layer added.

Through this bidirectional fusion mechanism, the P2 layer receives enriched semantic information from deeper layers via the top-down pathway, while its high-resolution spatial details are propagated back to deeper layers through the bottom-up pathway. This is particularly important for PWD detection. In the original image, early diseased trees occupying only a few pixels still retain sufficient spatial details on the P2 layer with a step size of 4, providing a basis for precise positioning. At the same time, the semantic features fused from layers P3 to P5 enhance the model’s recognition ability, enabling it to separate subtle symptoms from similar healthy backgrounds. **Figure 7** illustrates the overall feature extraction network with the integrated P2 detection layer.

### 2.4 Robust and lightweight feature modeling under complex backgrounds

Complex forest backgrounds introduce heavy interference, but practical deployment still requires high computational efficiency. We address both demands by optimizing the feature selection and feature fusion strategies together.

#### 2.4.1 EMA attention for background suppression

The C2f_DCNv4 module improves the network’s capacity to model geometric deformations that pine wilt disease targets exhibit, but diseased trees in complex forest environments are often intertwined with healthy vegetation, branches, and background elements—conditions under which feature representations become contaminated by background noise and detection accuracy suffers (Chen et al., 2025). Convolution-based extraction alone cannot reliably separate disease-relevant regions from visually similar surroundings, so an explicit feature reweighting mechanism is needed.

In the standard YOLOv8 backbone, the output feature map of the final stage is directly passed to the neck for multi-scale fusion. Denoting this feature map as $F\in {\mathbb{R}}^{C\times H\times W}$, the standard pipeline can be expressed as:

(7) $$F_{\text{out}}=\text{Neck}\left(F\right)$$

where each channel and spatial location contributes equally to subsequent detection stages, regardless of how relevant it is to the target. This uniform treatment is problematic for PWD detection, because disease-relevant features occupy only a small fraction of the feature map, while background regions dominate.

To address this, the Efficient Multi-scale Attention (EMA) mechanism is introduced between the backbone output and the neck input (Ouyang et al., 2023), transforming the pipeline into:

(8) $$F_{\text{out}}=\text{Neck}\left(\text{EMA}\left(F\right)\right)$$

The EMA module generates an attention weight map $A\in {\mathbb{R}}^{C\times H\times W}$ that reweights the input features, selectively amplifying disease-relevant activations while suppressing background responses. The reweighted feature can be expressed as Equation 9:

(9) $$F^{\prime }=A⊙F$$

where ⊙ denotes element-wise multiplication. The key distinction from Equation 7 is that each channel and spatial location now receives a differentiated weight based on its relevance to the detection target, rather than being treated uniformly.

To generate the attention weights, the EMA module first applies global average pooling across both spatial dimensions to capture channel-wise global context. For the c-th channel of the input feature map F (Equation 10):

(10) $$Z_{c}=\frac{1}{H\times W}\sum_{j=1}^{H}\sum_{i=1}^{W}x_{c}\left(i,j\right)$$

where *Z_c_* encodes the global spatial response of the *c*-th channel. Unlike standard channel attention methods such as SE (Hu et al., 2018), which directly use *Z_c_* to generate channel weights through dimensionality reduction, the EMA mechanism further divides the feature channels into multiple groups and applies parallel 1 × 1 and 3 × 3 convolutions within each group to capture both inter-channel dependencies and local spatial context. After the outputs of the two branches are merged along the spatial axis and activated by the Sigmoid function, the final attention weight map *A* is generated. This multi-scale grouping design avoids the bottleneck of traditional attention methods in dimensionality reduction, retains the fine-grained channel information, and this type of information is crucial for distinguishing the subtle feature differences between diseased and healthy pine trees at an early stage.

**Figure 8:**
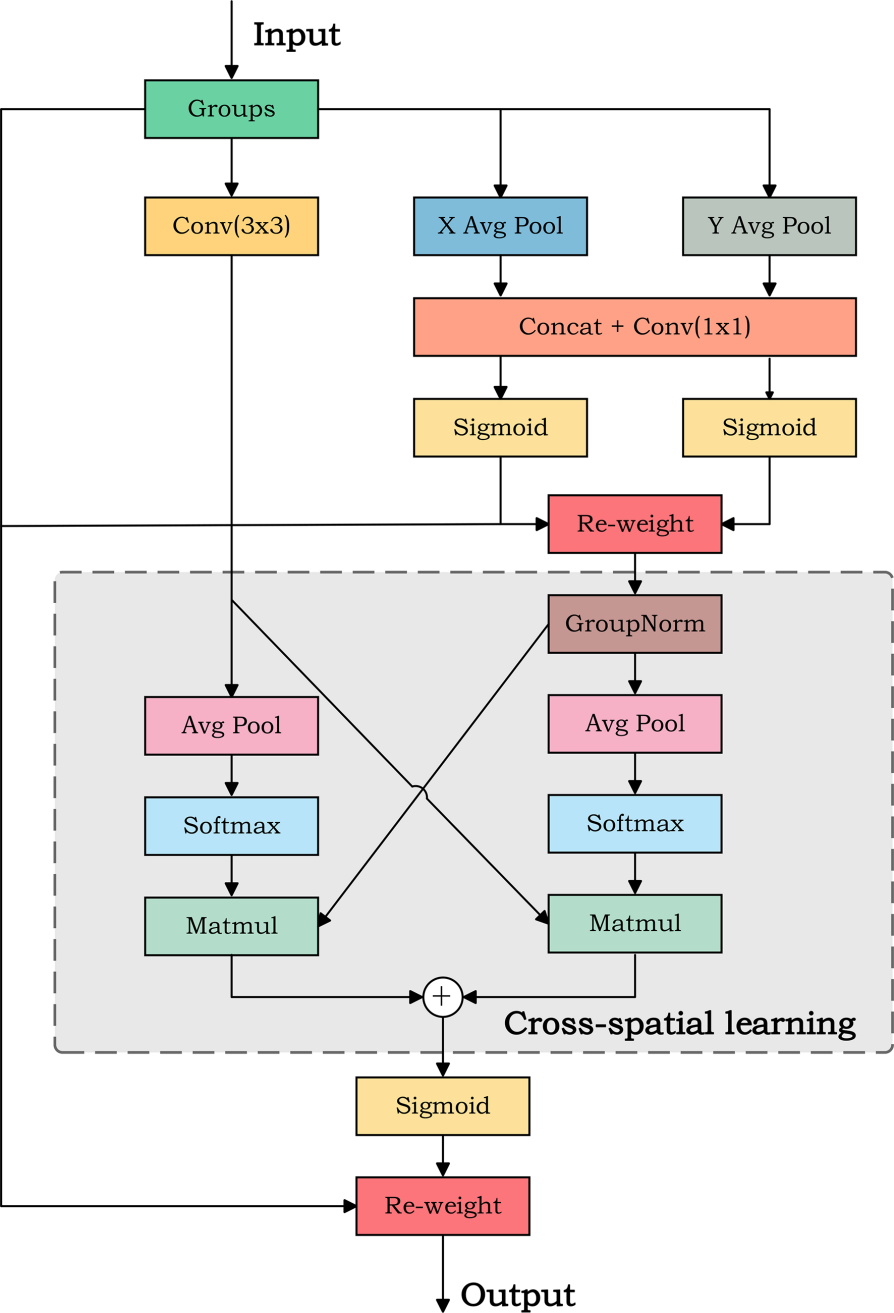
Structure of EMA attention mechanism.

A comparison of Equations 7, 8 shows that inserting EMA converts the backbone-to-neck connection from a passive pass-through into an active feature selection step. This matters for PWD detection because early-stage diseased trees generate weak feature responses that strong activations from surrounding healthy vegetation can easily overwhelm. By amplifying those weak disease signals and suppressing dominant background responses, the EMA module raises the signal-to-noise ratio of disease-relevant features before they enter the multi-scale fusion stage. As **Figure 8** shows, the module accomplishes this through grouped and parallel convolution operations that widen the receptive field while preserving spatial structure, keeping the architecture lightweight and suitable for further edge optimization.

#### 2.4.2 Efficient feature fusion via C2f_DualConv

In multi-scale feature fusion, the C2f module is widely used due to its efficient feature aggregation capability based on the Cross Stage Partial (CSP) strategy (Zhou et al., 2025). However, in pine wilt disease detection, forestry scenes are characterized by complex backgrounds, significant scale variation, and fine-grained details. The conventional C2f module mainly relies on a single convolutional structure, resulting in a relatively fixed receptive field and limited ability to represent multi-scale features (Khalili and Smyth, 2024). During feature fusion, a single convolution struggles to simultaneously capture local details and broader contextual information, restricting the model’s ability to effectively represent disease-related features.

**Figure 9:**
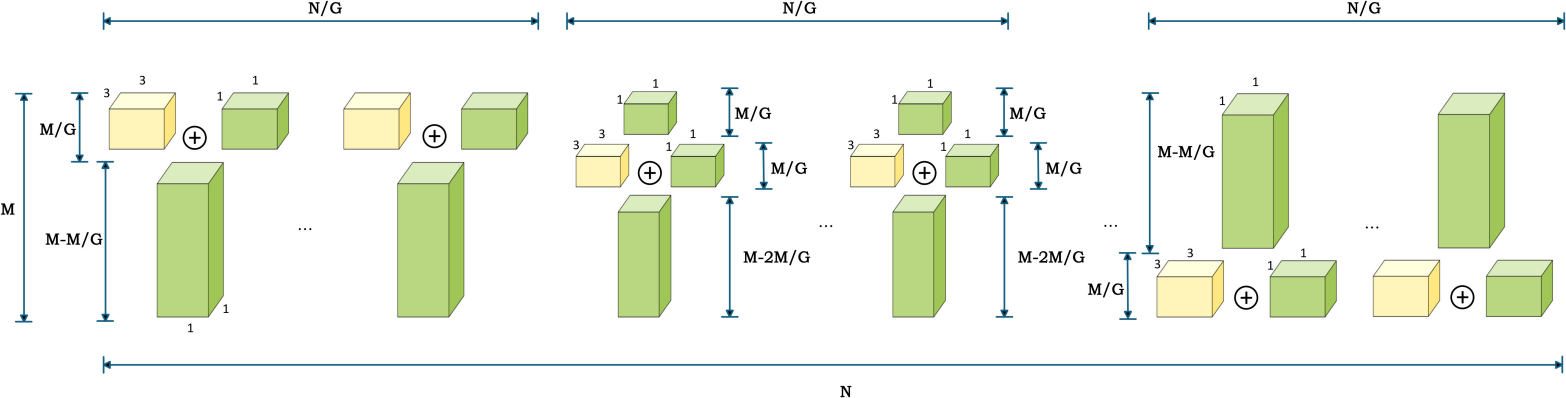
Filter design of DualConv.

As described in Section 2.3.1, the standard C2f block processes features through sequential Bottleneck modules with fixed-grid 3 × 3 convolutions (Equation 1). To enable simultaneous capture of local spatial details and broader channel-wise context within each Bottleneck, the standard convolution is replaced with a dual-branch structure (Zhong et al., 2023), constructing the C2f_DualConv module:

(11) $$F_{\text{C}2\text{f}\_\text{DualConv}}\left(X\right)={\text{Conv}}_{1\times 1}\left(\text{Concat}\left(X_{1},B_{1}^{\text{dual}}\left(X_{2}\right),B_{2}^{\text{dual}}\left(B_{1}^{\text{dual}}\left(X_{2}\right)\right),\ldots ,B_{N}^{\text{dual}}\left(\ldots \right)\right)\right)$$

where *B_i_*^dual^ denotes the modified Bottleneck with DualConv replacing the standard convolution. Within each *B_i_*^dual^, the input feature channels are divided into *G* groups, and two parallel convolution branches operate simultaneously (Equation 12):

(12) $$B_{i}^{\text{dual}}\left(F\right)=f_{\text{merge}}\left(f_{3\times 3}^{\text{group}}\left(F\right),f_{1\times 1}^{\text{point}}\left(F\right)\right)$$

where $f_{3\times 3}^{\text{group}}$ denotes grouped 3 × 3 convolution that captures local spatial context within each channel group, $f_{1\times 1}^{\text{point}}$ denotes 1 × 1 pointwise convolution that performs channel-wise feature reorganization, and *f*_merge_ represents the fusion of the two branches through channel concatenation. This dual-branch design imposes a block-diagonal sparsity pattern within the convolutional kernel matrix, prompting highly correlated channels to be processed collectively while reducing redundant computation. **Figure 9** illustrates the DualConv filter design, where *M* is the input channel count, *N* is the output channel count, and *G* is the number of groups used in the grouped convolution. Comparing Equations 1, 11, the central change is replacing the single-branch Bottleneck *B_i_* with the dual-branch variant *B_i_*^dual^. The standard C2f block captures features at one spatial scale per layer, whereas C2f_DualConv extracts multi-scale information within each Bottleneck simultaneously—local spatial patterns through grouped 3 × 3 convolution and global channel relationships through 1 × 1 convolution. This dual-scale design is especially valuable for PWD detection: the 3 × 3 branch captures spatial texture differences between diseased and healthy canopy at a local level, while the 1×1 branch models channel-wise spectral correlations that help distinguish disease-induced discoloration from natural color variation.

To quantitatively analyze the computational efficiency of this replacement, the floating-point operations (FLOPs) of standard convolution and DualConv are compared. Let the input feature map have a spatial size of *D*_0_ × *D*_0_, with *M* input channels and *N* output channels, and a convolution kernel size of *K* × *K*. The FLOPs of standard convolution can be expressed as:

(13) $${\text{FL}}_{\text{std}}=D_{0}^{2}\cdot K^{2}\cdot M\cdot N$$

For the DualConv structure, the total FLOPs consist of the grouped 3 × 3 branch and the 1 × 1 branch (Equations 14, 15):

(14) $${\text{FL}}_{\text{Dual}}^{3\times 3}=\frac{D_{0}^{2}\cdot K^{2}\cdot M\cdot N+D_{0}^{2}\cdot M\cdot N}{G}$$

(15) $${\text{FL}}_{\text{Dual}}^{1\times 1}=D_{0}^{2}\cdot M\cdot N\cdot \left(1-\frac{1}{G}\right)$$

Therefore, the total FLOPs of DualConv can be expressed as:

(16) $${\text{FL}}_{\text{Dual}}={\text{FL}}_{\text{Dual}}^{3\times 3}+{\text{FL}}_{\text{Dual}}^{1\times 1}=\frac{D_{0}^{2}\cdot K^{2}\cdot M\cdot N}{G}+D_{0}^{2}\cdot M\cdot N$$

By comparing Equations 13, 16, the computational reduction ratio of DualConv relative to standard convolution can be expressed as Equation 17:

(17) $$R=\frac{{\text{FL}}_{Dual}}{{\text{FL}}_{std}}=\frac{1}{G}+\frac{1}{K^{2}}$$

When *R<* 1, DualConv achieves lower computational cost than standard convolution. By appropriately selecting the group number *G* and kernel size *K*, significant reductions in FLOPs and parameter count can be achieved without substantially degrading feature representation capability. This efficiency gain is critical for maintaining the lightweight requirements of the overall MSDF-Net framework.

Through this design, the C2f_DualConv module is introduced into the multi-scale feature fusion stage to replace the original C2f structure, enhancing information interaction across different hierarchical levels during feature upsampling and fusion. The dual-branch Bottleneck enables the network to simultaneously integrate local spatial details and global channel context information at each fusion stage. This not only enhances the ability to distinguish PWD targets at different scales, but also keeps the computational cost at a sufficiently low level, making it a promising candidate for resource-constrained deployment scenarios.

## 3 Experiments and results

### 3.1 Experimental settings

The experimental platform was built on the PyTorch deep learning framework using the Ultralytics YOLOv8 implementation. The hardware environment and software configuration used in this study are summarized in **Table 2**, while the main training parameters of the model are listed in **Table 3**.

**Table 2:** Model training software and hardware environment configurations.

Software and hardware environment	Configuration
Operating system	Windows11
Software	Python3.10.0, PyTorch2.6.0, CUDA12.1
CPU	Intel(R) Core™ i7-14700KF @3.40GHz
GPU	NVIDIA GeForce RTX 4070 SUPER 12GB
RAM	32GB

**Table 3:** Training parameters.

Model training parameters	Configuration
Learning rate	0.01
Input image size	640×640
Epoch	200
Momentum	0.937
Batch size	8
Warmup bias lr	0.1
Weight decay	0.0005

All experiments were conducted under Windows 11. While Linux-based environments are the conventional standard for deep learning research, our experimental infrastructure was configured with Windows for compatibility with other forestry-related software tools in our research pipeline. The core operations used in our models—tensor computations, convolution routines, and CUDA kernel execution—are determined by the PyTorch 2.6.0 and CUDA 12.1 versions rather than the host operating system, and are therefore platform-agnostic at the computation level. To support reproducibility, our training protocol employed fixed random seeds across Python, NumPy, and PyTorch, which ensures run-to-run consistency on the same machine. We note, however, that fixed seeds do not by themselves guarantee bit-exact reproducibility across different operating systems, because non-deterministic CUDA kernels and differences in underlying BLAS libraries can introduce small numerical variations. We therefore expect our reported results to be reproducible to within small numerical tolerance under a standard Linux environment with the same PyTorch and CUDA versions, and we acknowledge that this expectation has not been empirically verified. Full reproduction under Linux is identified as a planned next step.

The model training parameters were primarily configured according to the official recommendations of YOLOv8 to ensure training stability and reproducibility (Yaseen, 2024). Meanwhile, key parameters such as batch size and the number of training epochs were further optimized through preliminary experiments. This parameter selection strategy is consistent with previous studies on similar object detection tasks (Li et al., 2024b).

**Figure 10:**
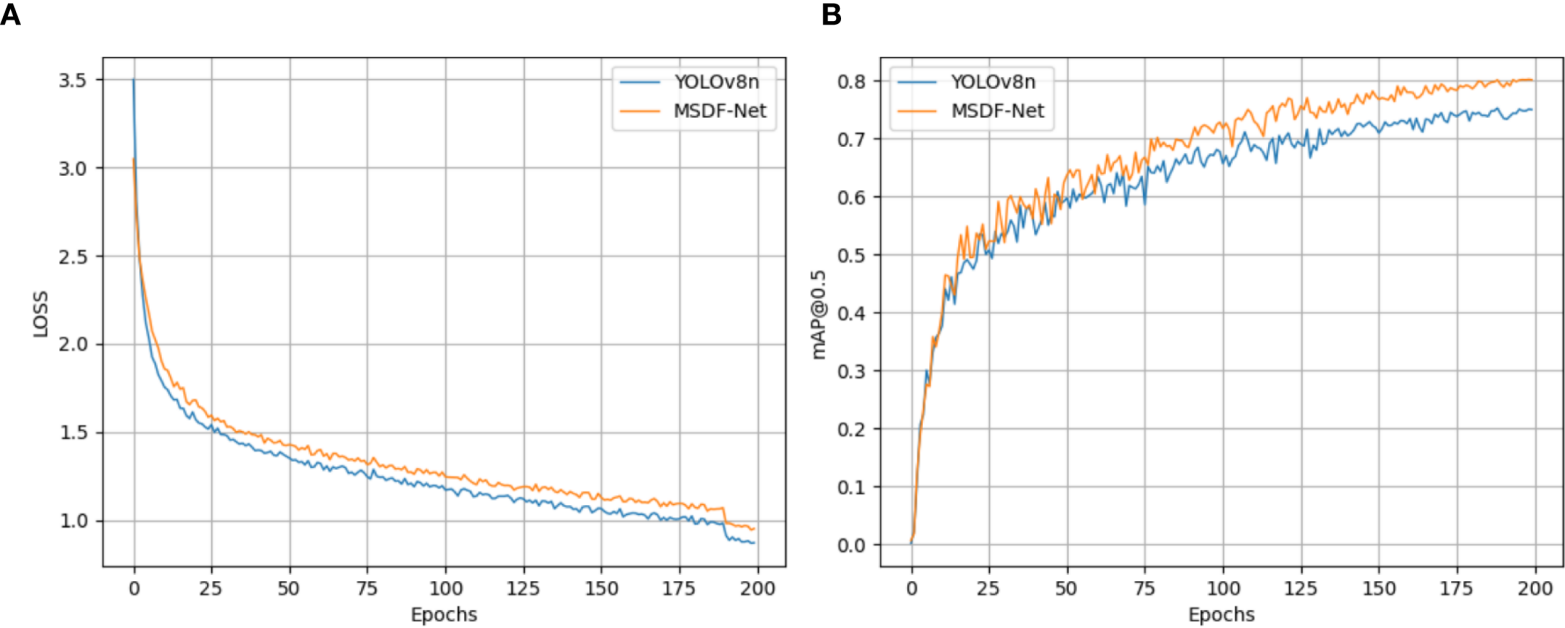
Training performance comparison between the baseline model and the proposed method: **(a)** training loss curves; **(b)** mAP@0.5 curves.

For fair comparison, the baseline detector (YOLOv8n) and the proposed method were trained under identical experimental conditions and parameter settings. The variations in training loss and mAP@0.5 during the training process are illustrated in **Figure 10**.

It is noteworthy that although MSDF-Net exhibits slightly higher training loss than the baseline YOLOv8n throughout the training process, it achieves higher mAP@0.5 in most training epochs. This pattern indicates that MSDF-Net achieves superior detection performance despite a slightly higher training loss, which is consistent with stronger generalization rather than tighter training-set fitting.

### 3.2 Evaluation metrics

We evaluated the MSDF-Net under the same experimental conditions from two different aspects. In the ablation experiments, we used precision, recall rate, and the mean average precision (mAP) to assess the detection performance, and then analyzed the contribution of each module. In the cross-version transfer verification, we focused on the mAP of the overall model and each category, as well as the complexity indicators of the model.

Precision measures prediction accuracy—the fraction of true positive identifications out of all instances the model classifies as positive. Recall measures the model’s ability to find targets—the proportion of correctly detected targets among all ground truth instances. Average Precision (AP) is the area under the Precision-Recall curve, which plots how precision varies across different recall levels, and it summarizes detection performance for a single class. In multi-class tasks, Mean Average Precision (mAP) averages the AP values across all classes to give an overall measure of detection capability. When the IoU threshold is set to 0.5, this metric is written as mAP@0.5. In addition to the detection accuracy indicators, we also measure the model size by the number of parameters (Parameters) and the computational cost by the number of floating-point operations (FLOPs).

The mathematical formulations of the above evaluation metrics are as follows (Equations 18–21):

(18) $$P=\frac{TP}{TP+FP}$$

(19) $$R=\frac{TP}{TP+FN}$$

(20) $$AP=\int_{0}^{1}P\left(R\right)dR$$

(21) $$mAP=\frac{1}{C}\sum_{i=1}^{C}AP_{i}$$

Here, True Positive (TP) is the number of instances the model correctly identifies as targets; False Positive (FP) is the number of background samples the model misclassifies as targets; and False Negative (FN) is the number of actual targets the model fails to detect. *C* is the total number of object classes in the dataset, and *AP_i* is the average precision for the *i*-th class.

In addition to detection accuracy metrics, this study also evaluates the proposed method from the perspective of model complexity. Specifically, the number of model parameters (Parameters) is used as an indicator of spatial complexity, reflecting the model size, while the number of floating point operations (Floating Point Operations, FLOPs) is adopted as an indicator of computational complexity, measuring the amount of computation required during inference. By jointly considering detection accuracy and model complexity, a more thorough evaluation of the proposed method can be obtained for practical forestry monitoring applications.

### 3.3 Experimental validation of model components

To evaluate the contributions of each improvement module, we conducted ablation experiments using YOLOv8n as the baseline. Under the same training strategy, data division, and hyperparameter settings, we added each module one by one and tested different combinations. The results are shown in **Table 4**, and the symbol “ √“ indicates that the corresponding module is included in that configuration.

**Table 4:** Ablation experiment results.

Baseline	C2f DCNv4	EMA	C2f DualConv	P2	Precision	Recall	mAP@0.5	Parameters	FLOPs
YOLOv8n	–	–	–	–	72.2%	70.1%	75.0%	3.01M	8.1G
YOLOv8n	✓	–	–	–	73.1%	72.0%	74.9%	2.91M	8.0G
YOLOv8n	–	✓	–	–	78.5%	67.7%	74.2%	3.04M	8.2G
YOLOv8n	–	–	✓	–	72.3%	70.1%	75.1%	2.86M	7.8G
YOLOv8n	–	–	–	✓	73.6%	73.0%	78.0%	2.92M	12.2G
YOLOv8n	✓	✓	–	–	73.4%	73.1%	75.8%	2.91M	8.0G
YOLOv8n	✓	–	✓	–	74.6%	69.6%	74.8%	2.76M	7.7G
YOLOv8n	✓	–	–	✓	71.9%	73.3%	78.0%	2.82M	12.1G
YOLOv8n	–	✓	✓	–	75.0%	70.8%	76.0%	2.86M	7.8G
YOLOv8n	–	✓	–	✓	77.0%	71.8%	78.9%	2.96M	12.4G
YOLOv8n	–	–	✓	✓	68.5%	76.2%	78.6%	2.77M	11.8G
YOLOv8n	✓	✓	✓	–	80.8%	66.4%	76.3%	2.76M	7.7G
YOLOv8n	✓	✓	–	✓	71.6%	74.9%	79.7%	2.86M	12.3G
YOLOv8n	✓	–	✓	✓	73.3%	73.7%	78.8%	2.67M	11.7G
YOLOv8n	–	✓	✓	✓	72.9%	72.4%	79.2%	2.81M	12.0G
YOLOv8n	✓	✓	✓	✓	73.2%	74.6%	80.1%	2.67M	11.7G

When each module is introduced separately, the impact on performance varies. C2f DCNv4 increases the accuracy from 72.2% to 73.1% and the recall rate from 70.1% to 72.0%, indicating an improvement in feature extraction. However, mAP@0.5 drops to 74.9%. EMA increases the accuracy to 78.5% but the recall rate drops to 67.7%. This result suggests that the model suppresses background interference while failing to detect some low-confidence targets. C2f_DualConv increases mAP@0.5 to 75.1% while reducing the computational cost, confirming its effectiveness in lightweight optimization. The P2 detection layer, although increasing FLOPs to 12.2G, raises the recall rate to 73.0% and the mAP@0.5 to 78.0%, highlighting its advantage in perceiving small objects.

The non-monotonic effects of C2f_DCNv4 and EMA can be explained by analyzing how each module influences the distribution of feature responses. For C2f_DCNv4, the learnable spatial offsets enhance sensitivity to irregular disease boundaries, thereby improving both precision and recall. However, in the absence of a gating mechanism, the offsets occasionally drift to visually similar background regions, producing a small number of high-confidence false positives. Because mAP@0.5 integrates precision across all confidence levels, these few errors disproportionately lower the overall metric despite the broad improvements. In essence, C2f_DCNv4 strengthens feature representation but simultaneously increases response variance, and the net effect on mAP can be marginally negative. For EMA, the mechanism is different. When EMA is applied to features extracted by standard fixed-grid convolutions, the feature signals of early-stage disease targets are already weak and spatially diffuse. The attention module, while effectively suppressing background noise, inadvertently attenuates some genuine low-confidence targets as well, resulting in higher precision but lower recall. When the two modules are combined, DCNv4 first strengthens and sharpens target features, giving EMA a more reliable basis for distinguishing disease signals from background; EMA then suppresses noise without accidentally eliminating weak targets, allowing the improved representation to translate into a net mAP gain. This functional complementarity—where DCNv4 reduces feature variance and EMA provides feature selection—justifies both the module design and the insertion order: DCNv4 is placed at the deepest backbone layer for reliable offset learning, and EMA is positioned at the backbone-to-neck interface for gating before multi-scale fusion.

Combining modules reveals both complementary and competitive interactions. C2f DCNv4 paired with EMA, for example, pushes recall to 73.1% and mAP@0.5 to 75.8%, which shows the two modules reinforcing each other during feature enhancement. C2f_DCNv4 together with C2f_DualConv keeps detection performance stable while trimming both parameters and FLOPs—an efficient lightweight combination. When EMA is added alongside the P2 detection layer, mAP@0.5 climbs further to 78.9%, pointing to clear synergy in detection accuracy.

Multi-module integration brings trade-offs as well. EMA combined with C2f_DualConv raises recall to 76.2% but drops precision to 68.5%, which means more false positives. Similarly, stacking C2f_DCNv4, EMA, and C2f_DualConv together lifts precision to 80.8% while recall falls to 66.4%—a classic precision–recall trade-off.

With all four components integrated, the proposed model reaches its best overall performance: 73.2% precision, 74.6% recall, and 80.1% mAP@0.5. Relative to the baseline, mAP@0.5 gains 5.1 percentage points and recall gains 4.5 percentage points, while parameters drop from 3.01M to 2.67M and FLOPs rise modestly to 11.7G. Each module contributes a distinct strength: C2f_DCNv4 strengthens feature extraction, EMA sharpens the model’s focus on key regions, C2f_DualConv enables lightweight optimization, and the P2 detection layer boosts small-object detection. Together they produce a favorable trade-off between detection precision and computational cost, confirming that the proposed approach is both effective and practically valuable.

To further verify that the performance gains from the proposed modules are not merely attributable to increased model capacity, we compared MSDF-Net with a scaled-up baseline variant, YOLOv8s, trained under identical conditions. The results are presented in **Table 5**. Scaling from YOLOv8n to YOLOv8s yields a gain of only 2.7 percentage points in mAP@0.5 at the cost of a 3.7× increase in parameters and a 3.5× increase in FLOPs. In contrast, MSDF-Net achieves a gain of 5.1 percentage points over YOLOv8n while reducing parameters by 11.3% (3.01M to 2.67M). The difference is even more pronounced for early-stage detection: YOLOv8s improves PWD-E AP by only 2.7 percentage points over YOLOv8n (39.2% to 41.9%), whereas MSDF-Net improves it by 20.2 percentage points (39.2% to 59.4%). This confirms that simply increasing model depth and width cannot address the fundamental challenges of small-target and irregular-shape detection that our targeted architectural modifications—particularly the P2 detection layer and DCNv4 deformable convolution—are designed to solve.

**Table 5:** Comparison with scaled-up YOLOv8s under identical training.

Model	Parameters	FLOPs	mAP@0.5	PWD-E AP
YOLOv8n	3.01M	8.1G	75.0%	39.2%
YOLOv8s	11.13M	28.4G	77.7%	41.9%
MSDF-Net	2.67M	11.7G	80.1%	59.4%

Beyond the accuracy–capacity trade-off, we also characterized the practical efficiency of each module by measuring per-module FLOPs, peak GPU memory consumption, and end-to-end inference latency for three key configurations: the baseline YOLOv8n with three detection heads, YOLOv8n with the P2 layer added (four heads), and the full MSDF-Net. The results are summarized in **Table 6**. From the per-module perspective, the FLOPs contributions can be isolated from **Table 4**: C2f_DCNv4 reduces FLOPs by 0.1G (8.1G to 8.0G) due to its efficient grouped deformable implementation; EMA adds only 0.1G (8.1G to 8.2G); and C2f_DualConv reduces FLOPs by 0.3G (8.1G to 7.8G) through its dual-branch design. The P2 detection head is the dominant source of additional computation, adding 4.1G FLOPs, because the P2 feature map at 160 × 160 spatial resolution contains four times as many grid cells as P3 (80 × 80), proportionally increasing the number of candidate proposals processed by the detection head.

**Table 6:** Per-configuration efficiency analysis.

Configuration	Params (M)	FLOPs (G)	GPU memory (MB)	Inference time (ms)
YOLOv8n (3 heads)	3.01	8.1	35.2	10.13
YOLOv8n + P2 (4 heads)	2.92	12.2	68.0	13.46
MSDF-Net (full, 4 heads)	2.67	11.7	120.4	16.99

As shown in **Table 6**, adding the P2 layer increases inference time from 10.13 ms to 13.46 ms (+33%) and peak GPU memory from 35.2 MB to 68.0 MB (+93%). The full MSDF-Net further increases inference time to 16.99 ms (+68% over baseline) and peak GPU memory to 120.4 MB. This additional cost is primarily attributable to two sources: the increased number of raw candidate proposals from the P2 layer (approximately 25,600 additional grid cells at stride 4, increasing the total proposal count by roughly a factor of four compared with the three-head configuration) and the computational overhead of the DCNv4 deformable convolution and EMA attention modules. Despite these increases, the absolute inference latency of 16.99 ms (approximately 59 FPS) remains well within the throughput requirements of UAV-based monitoring, where image capture intervals are typically on the order of seconds, and the 120.4 MB peak memory footprint suggests the model can plausibly fit within the unified memory budget of modern edge devices such as the NVIDIA Jetson Orin NX (8–16 GB shared CPU/GPU memory). We note, however, that these benchmarks were conducted on a desktop GPU (RTX 4070 SUPER) rather than on actual edge hardware, and that desktop peak memory and edge unified memory are not directly comparable. On-device profiling with TensorRT optimization therefore remains a necessary next step toward deployment.

To interpret these improvements further, we carry out a visualization analysis grounded in the ablation results. We use the XGrad-CAM method to visualize detection outputs and compare how their responses differ across target regions (Fu et al., 2020).

**Figure 11:**
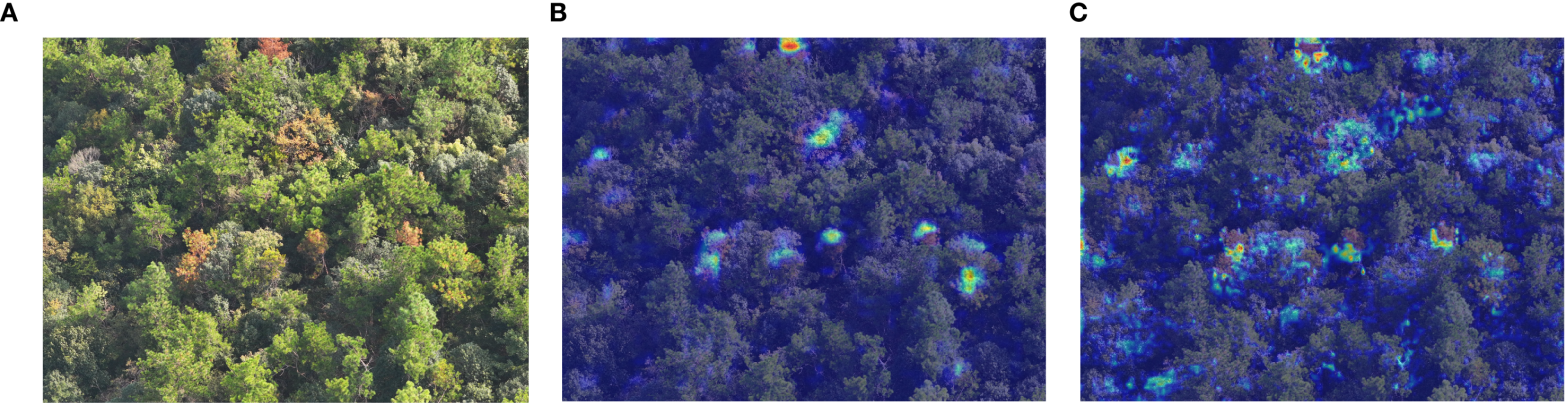
Heatmap comparison of different models for pine wilt disease detection. **(a)** Input image; **(b)** YOLOv8n; **(c)** MSDF-Net.

The visualization results appear in **Figure 11**. Heatmap responses from the original YOLOv8 model show a noticeable diffusion effect: boundaries are blurred and scattered pseudo-responses appear across the healthy-tree background, which can interfere with target recognition and cause missed detections. The improved model, by contrast, produces more compact and sharply defined heatmap responses that follow the canopy contours of individual diseased trees closely. At the same time, background noise from healthy vegetation is significantly suppressed, leading to a substantial improvement in contrast between target and background regions. The heat intensity is more concentrated within the diseased canopy areas, effectively enhancing the localization accuracy and recognition robustness of the model for pine wilt diseased trees, thereby better satisfying the practical requirements of forestry remote sensing applications.

### 3.4 Module transferability validation

As discussed in the introduction, the YOLO family exhibits significant structural differences across generations—YOLOv8 uses C2f blocks, while more recent variants such as YOLOv11, YOLOv12, and YOLOv13 adopt C3k2-based blocks with distinct internal routing. Modules tightly coupled to a specific block structure, such as C2f_DualConv and C2f_DCNv4, which modify the internal split-concatenate logic of C2f, cannot be directly ported to architectures using C3k2-based blocks. We confirmed this incompatibility empirically: substituting C2f_DualConv or C2f_DCNv4 for the corresponding C3k2 blocks in the YOLOv11n/12n/13n configuration files produced runtime tensor-shape mismatch errors during the forward pass, because the C3k2 block exposes a different intermediate-channel and split signature than C2f, and its internal C3k submodule wires its bottlenecks differently from C2f’s bottleneck path. A faithful port would therefore require redesigning the modules at the C3k2 block-internal level (e.g., constructing analogous C3k2_DualConv and C3k2_DCNv4 variants), which is a substantive engineering effort beyond a simple substitution and is identified as future work below. In contrast, DCNv4 functions as a replacement for any standard 3 × 3 convolution regardless of the surrounding block structure, and the P2 detection layer is an addition to the detection head that is independent of the backbone design. EMA, which operates at the backbone-to-neck interface through channel grouping and cross-spatial attention, is also structurally independent of the C2f/C3k2 block design and is transferable in principle. However, the cross-version validation in this study is deliberately scoped to DCNv4 and P2, the two modules that address the most fundamental PWD detection challenges—irregular shape modeling and small-target sensitivity—and contribute the majority of the observed improvements. EMA’s contribution, while valuable, is context-dependent and secondary: as shown in **Table 4**, EMA provides a modest gain only when paired with DCNv4, which first strengthens the upstream features that EMA relies on for reliable gating. Including EMA in the cross-version transferability table without per-architecture verification of this DCNv4–EMA interaction would risk overstating its independent transferability. Therefore, the full MSDF-Net framework (C2f_DCNv4, C2f_DualConv, EMA, and P2) is implemented on YOLOv8n as the primary architecture, while DCNv4 and P2 are further validated on YOLOv11n, YOLOv12n, and YOLOv13n. Investigating EMA’s cross-version behavior—together with adapting C2f_DualConv and C2f_DCNv4 to C3k2-style blocks—is a direction for future work.

It is worth noting that baseline mAP@0.5 decreases from YOLOv8n (75.0%) to YOLOv11n (73.4%), YOLOv12n (71.2%), and YOLOv13n (68.9%), despite these being more recent architectures. This is because newer YOLO variants introduce increasingly compact and specialized designs that are primarily optimized for large-scale general-purpose benchmarks such as COCO. In domain-specific forestry scenarios where targets are small, irregularly shaped, and sparsely distributed, these architectural innovations do not necessarily translate into performance gains. This further justifies the selection of YOLOv8n as the primary base architecture, as its C2f structure provides stronger general-purpose feature extraction suited to the characteristics of this task. Reading **Table 7** against this baseline trend makes the consistent positive gains achieved by our modules across all four versions even more meaningful.

**Table 7:** Module transferability.

Model	mAP@0.5	ΔmAP	Parameters	FLOPs
YOLOv8n	75.0%	–	3.01M	8.1G
YOLOv8n+MSDF-Net	80.1%	+5.1%	2.67M	11.7G
YOLOv11n	73.4%	–	3.70M	11.0G
YOLOv11n+DCNv4+P2	79.5%	+6.1%	3.80M	11.3G
YOLOv12n	71.2%	–	2.55M	6.5G
YOLOv12n+DCNv4+P2	75.5%	+4.3%	2.38M	12.3G
YOLOv13n	68.9%	–	2.46M	6.3G
YOLOv13n+DCNv4+P2	74.0%	+5.1%	2.32M	12.8G

The transferability validation results are presented in **Table 7**. On YOLOv8n, the integration of the full MSDF-Net module set (DCNv4, P2, EMA, and C2f_DualConv) improved mAP@0.5 from 75.0% to 80.1%, a gain of 5.1 percentage points, while reducing parameters from 3.01M to 2.67M. On YOLOv11n, integrating only DCNv4 and P2 yielded a 6.1 percentage point improvement from 73.4% to 79.5%. On YOLOv12n, the same two modules improved mAP@0.5 from 71.2% to 75.5%, a gain of 4.3 percentage points, while reducing parameters from 2.55M to 2.38M. On YOLOv13n, the integration achieved a 5.1 percentage point improvement from 68.9% to 74.0%, again with a slight parameter reduction from 2.46M to 2.32M. Although the FLOPs increase from 6.5G and 6.3G to 12.3G and 12.8G on YOLOv12n and YOLOv13n respectively due to the added P2 detection layer at higher spatial resolution, the consistent positive gains across four architecturally distinct YOLO generations—each with a weaker starting baseline than the last—confirm that the proposed modules address fundamental limitations in small-object detection and deformable feature modeling rather than exploiting architecture-specific characteristics.

To further analyze the impact of the proposed modules on different disease severity levels, per-class AP@0.5 results are compared in **Table 8**. The most substantial improvement was consistently observed in the PWD-E (early-stage) category across all four architectures. On YOLOv8n, the accuracy rate of PWD-E detection increased from 39.2% to 59.4% (20.2 percentage points). On YOLOv11n, the accuracy rate of PWD-E detection rose from 38.1% to 55.5% (17.4 percentage points). On YOLOv12n and YOLOv13n, PWD-E improved from 33.5% to 52.7% (19.2 percentage points) and from 31.5% to 46.8% (15.3 percentage points), respectively.

**Table 8:** Per-class mAP@0.5 comparison.

Class	YOLOv8n	YOLOv8n +MSDF-Net	YOLOv11n	YOLOv11n +DCNv4+P2	YOLOv12n	YOLOv12n +DCNv4+P2	YOLOv13n	YOLOv13n +DCNv4+P2
PWD-E	39.2%	59.4%	38.1%	55.5%	33.5%	52.7%	31.5%	46.8%
PWD-M	82.5%	81.8%	77.2%	82.1%	74.6%	77.7%	73.2%	76.7%
PWD-L	95.0%	93.9%	94.6%	95.7%	93.2%	94.3%	93.1%	93.0%
PWD-D	83.4%	85.3%	83.7%	84.8%	83.6%	77.5%	77.7%	79.5%

For the remaining categories, improvements were also observed across most architectures. PWD-M detection improved consistently, with gains of 4.9, 3.1, and 3.5 percentage points on YOLOv11n, YOLOv12n, and YOLOv13n respectively, while maintaining comparable performance on YOLOv8n (82.5% vs. 81.8%). The PWD-L stage, which is characterized by more distinct visual features, consistently maintained a high AP value (over 93%) across all models. For PWD-D, improvements were observed on YOLOv8n, YOLOv11n, and YOLOv13n, while a drop from 83.6% to 77.5% occurred on YOLOv12n. This isolated regression is most plausibly explained by a specific architectural interaction unique to YOLOv12n. Among the four YOLO variants tested, only YOLOv12n employs area-based attention within its C3k2 blocks: feature maps are partitioned into spatial regions and attention is computed locally within each region. This design biases the network toward fine-grained local features, which benefits small targets but introduces a side effect when the high-resolution P2 detection layer is added. With four detection scales (P2–P5), the bidirectional FPN+PAN fusion propagates the spatially detailed but semantically weak P2 features upward through the bottom-up pathway into P4 and P5. Under YOLOv12n’s area-based attention, these fine-grained P2-derived features generate stronger local activation responses than the smoother, more globally distributed features that characterize large PWD-D targets at deeper layers. Consequently, the high-level semantic features critical for discriminating large dead-tree crowns are partially diluted, selectively degrading PWD-D accuracy. This effect is specific to YOLOv12n’s attention mechanism and does not occur in YOLOv8n (standard convolutions), YOLOv11n (C3k2 without area-based attention), or YOLOv13n (refined C3k2 with different routing). Nevertheless, the overall mAP@0.5 on YOLOv12n still improved by 4.3 percentage points, and the 19.2 percentage point gain in PWD-E far outweighs the 6.1 percentage point loss in PWD-D in practical disease-management terms, since early detection is the prerequisite for timely intervention.

These results demonstrate that the proposed modules serve as effective plug-and-play enhancements for YOLO-based detection frameworks, consistently improving overall detection performance—particularly for early-stage and small-scale targets—across multiple generations of the YOLO family. This improvement has been consistently demonstrated across the four YOLO versions YOLOv8, YOLOv11, YOLOv12, and YOLOv13, which differ substantially in their internal block design (C2f vs. C3k2) and feature-fusion routing. This cross-version generalization within the YOLO family strongly suggests that the proposed design captures fundamental properties of small-target and irregular-shape detection rather than artifacts of any single YOLO version, and that its application value is not limited to a specific backbone version. We note, however, that validation in this study is intentionally restricted to the YOLO family of single-stage anchor-free detectors. Transferability to architecturally distinct paradigms—such as two-stage detectors (e.g., Faster R-CNN) or transformer-based detectors (e.g., RT-DETR, DETR)—is not evaluated here and is identified as future work below.

### 3.5 Detection results and robustness analysis

**Figure 12:**
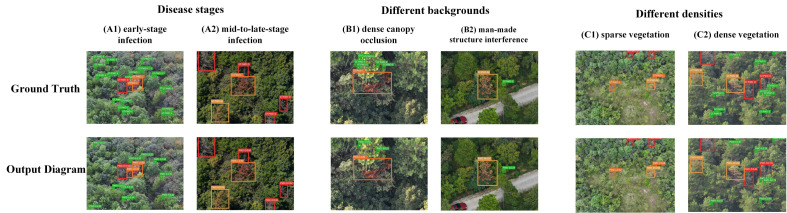
Detection results of MSDF-Net under different conditions. Disease stages: (A1) early-stage infection, (A2) mid- to late-stage infection; Different backgrounds: (B1) dense canopy occlusion, (B2) man-made structure interference; Different target densities: (C1) sparse vegetation, (C2) dense vegetation. Top row: ground truth annotations; Bottom row: MSDF-Net predictions.

To visually evaluate the detection performance and robustness of the model in different real-world scenarios, we present the visual results from six representative scenarios in **Figure 12**. These scenarios are organized along three dimensions, corresponding to the core detection challenges addressed in this paper: disease stage (A1 early-stage infection, A2 mid-to-late-stage infection), background type (B1 dense canopy occlusion, B2 man-made structure interference), and target density (C1 sparse targets, C2 dense targets). Within each sub-class, the upper part shows the real annotations, and the lower part shows the corresponding predictions of MSDF-Net. The color of the bounding boxes indicates the severity of the disease: green represents PWD-E, yellow represents PWD-M, orange represents PWD-L, and red represents PWD-D.

Along the disease stage dimension, comparing panels (A1) and (A2) shows how the model handles targets with different levels of visual prominence. In (A1), the scene contains many small PWD-E targets with subtle color changes distributed within dense canopy; MSDF-Net recovers most early-stage infections with reasonable confidence scores, addressing Problem 1—insufficient feature representation for small-scale and irregular targets. In (A2), visually distinct late-stage targets (PWD-L and PWD-D) dominate the scene, and the model maintains accurate localization and classification with high confidence, confirming that the enhancements for small-target detection do not compromise performance on larger targets.

Panels (B1) and (B2) examine robustness against two types of background interference. In (B1), the healthy canopy part partially blocks the affected area. The MSDF-Net not only successfully locates the PWD-L targets that are obscured, but also captures the surrounding small PWD-E infected areas, thanks to the adaptive spatial sampling mechanism of DCNv4 and the high-resolution features provided by the P2 detection layer. In (B2), roads and vehicles and other artificial buildings and facilities serve as non-vegetation interference sources, breaking the conventional composition of the forest background. Despite this interference, the MSDF-Net still correctly identifies the PWD-M and PWD-E targets, without generating false detections in non-vegetation areas, and demonstrates strong feature recognition ability under complex real conditions.

Panels (C1) and (C2) test model behavior at opposite ends of target density. (C1) presents a scene with sparse vegetation, where the targets are scattered and the background interference is minimal. The model accurately locates and classifies PWD-L and PWD-M targets with high confidence and reliable class assignment. This confirms that when the target distribution is sparse and visually prominent, MSDF-Net maintains stable and reliable discrimination performance. (C2) packs multiple diseased trees covering all severity levels—PWD-E, PWD-M, PWD-L, and PWD-D—into a single frame. MSDF-Net detects most of these targets and assigns the correct severity class to each, handling a dense multi-target scenario where conventional detectors often miss detections or confuse class labels.

To complement the qualitative visual analysis, we further quantified detection performance on each of the six representative scenarios independently. A test image may belong to more than one scenario when multiple challenge dimensions coexist. The per-scenario precision, recall, and mAP@0.5 values for both MSDF-Net and the baseline YOLOv8n are reported in **Table 9**.

**Table 9:** Per-scenario detection performance of YOLOv8n and MSDF-Net.

Scenario	Model	Precision	Recall	mAP@0.5
A1 (Early-stage)	YOLOv8n	76.8%	72.6%	78.7%
A1 (Early-stage)	MSDF-Net	77.9%	80.6%	85.4%
A2 (Mid-to-late)	YOLOv8n	70.3%	71.4%	75.5%
A2 (Mid-to-late)	MSDF-Net	76.7%	70.9%	77.8%
B1 (Occlusion)	YOLOv8n	67.4%	72.6%	71.8%
B1 (Occlusion)	MSDF-Net	76.4%	66.1%	79.1%
B2 (Man-made)	YOLOv8n	74.2%	66.4%	74.5%
B2 (Man-made)	MSDF-Net	77.0%	66.0%	75.7%
C1 (Sparse)	YOLOv8n	66.8%	58.6%	66.3%
C1 (Sparse)	MSDF-Net	73.2%	62.5%	76.6%
C2 (Dense)	YOLOv8n	75.3%	73.1%	77.7%
C2 (Dense)	MSDF-Net	76.9%	75.7%	83.1%

Scenarios A1/A2 (disease stage), B1/B2 (background type), and C1/C2 (target density) are defined in **Figure 12**.

These quantitative results provide numerical support for the visual patterns observed in **Figure 12**. The most substantial improvement occurs in the early-stage scenario A1, where MSDF-Net achieves an mAP@0.5 of 85.4%, a gain of 6.7 percentage points over YOLOv8n (78.7%), driven primarily by the large recall improvement (72.6% to 80.6%) from the P2 high-resolution detection layer. In the dense multi-target scenario C2, MSDF-Net attains 83.1% mAP@0.5, a gain of 5.4 percentage points. Under dense canopy occlusion (B1), MSDF-Net reaches 79.1%, a gain of 7.3 percentage points, reflecting the combined effect of EMA background suppression and DCNv4 adaptive spatial sampling. The sparse scenario C1 shows the largest relative gain of 10.3 percentage points (66.3% to 76.6%), confirming that the architectural enhancements generalize well to sparse-target conditions. The mid-to-late scenario A2 and the man-made interference scenario B2 show modest improvements of 2.3 and 1.2 percentage points respectively, indicating that the enhancements for challenging small and occluded targets do not compromise performance on visually distinct targets or non-vegetation backgrounds. Moreover, the standard deviation of mAP@0.5 across the six scenarios is 3.50 percentage points for MSDF-Net, compared to 4.13 percentage points for YOLOv8n. The reduced variance quantitatively demonstrates that MSDF-Net achieves more stable detection performance across diverse real-world conditions.

**Figure 13:**
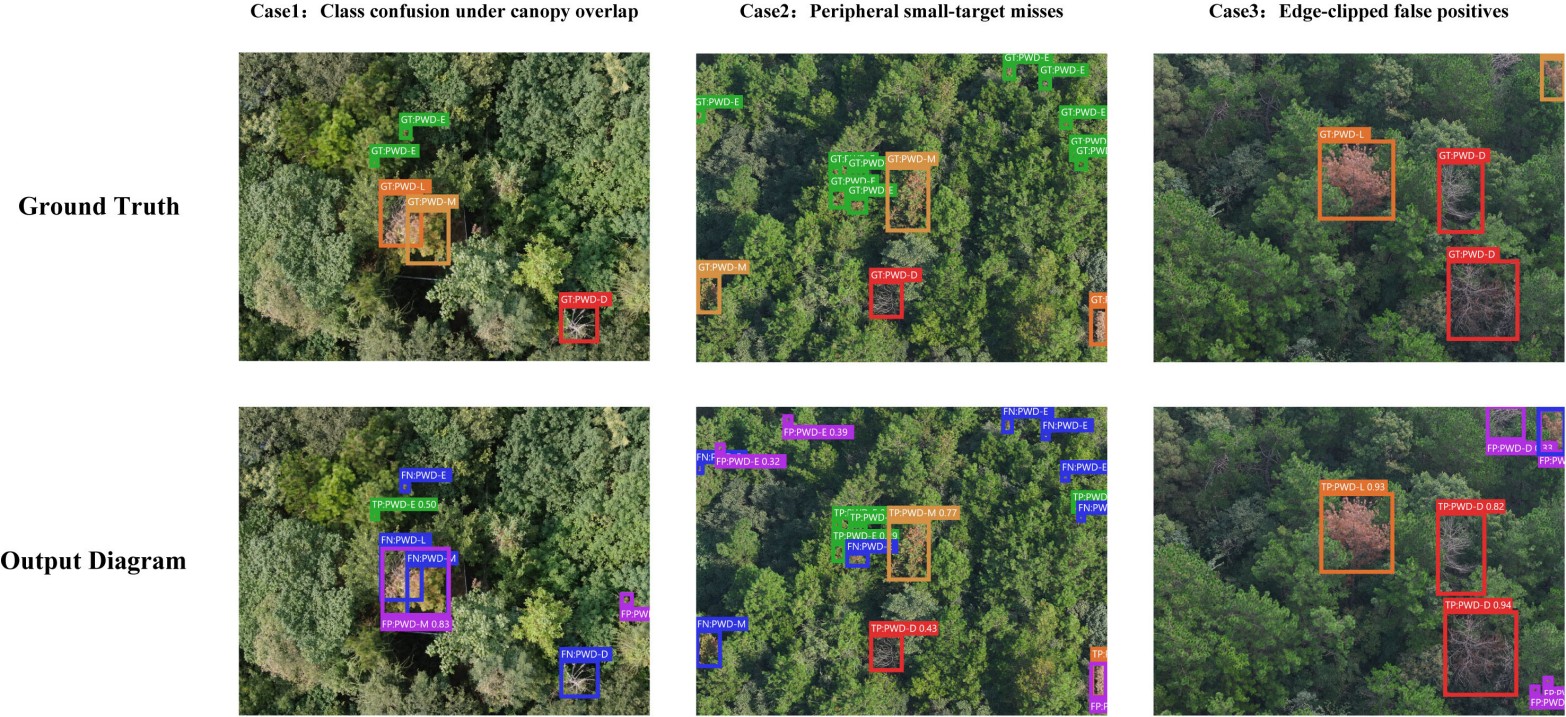
Representative failure cases of MSDF-Net. Top: ground truth; bottom: predictions. Case 1: class confusion under canopy overlap. Case 2: boundary false negatives and false positives. Case 3: edge-clipped false positives. Blue: FN; purple: FP; green/yellow/orange/red: TP for PWD-E/M/L/D.

To provide a more balanced assessment, we further examined the cases in which MSDF-Net still fails. Three representative examples, selected to cover distinct error patterns rather than to portray uniformly weak performance, are shown in **Figure 13**. In each pair, the top image shows the ground-truth annotation and the bottom image shows the model prediction, with false negatives drawn in blue, false positives in purple, and true positives in the same color scheme as **Figure 12**.

Case 1 illustrates a class-confusion failure under canopy overlap. Two adjacent diseased trees—one in the PWD-L stage and one in the PWD-M stage—are visually intertwined within a single crown cluster, and the model collapses them into a single PWD-M detection at 0.83 confidence rather than separating them. A nearby PWD-D tree at the lower-right corner and one of the small PWD-E targets are also missed. This pattern points to a limitation of the current bounding-box formulation when stage boundaries are spatially continuous rather than discrete: even with the DCNv4 deformable sampling, the network favors a single dominant prediction over two overlapping but semantically distinct ones.

Case 2 illustrates the residual difficulty of PWD-E detection in dense canopies. While several central PWD-E and PWD-M targets are correctly recovered, multiple peripheral PWD-E targets near the image boundary are missed, and the model also produces two low-confidence PWD-E false positives (0.32 and 0.39) in regions of healthy canopy with subtle off-green color variation. The P2 detection layer and DCNv4 substantially improve PWD-E recall overall (**Table 9**), but the boundary between genuine early chlorosis and natural canopy heterogeneity remains inherently ambiguous at the few-pixel scale, and this ambiguity is what residual false positives and false negatives both reflect.

Case 3 illustrates an edge-clipped false-positive pattern in an otherwise easy scene. The three main targets in the center of the frame—one PWD-L and two PWD-D trees—are detected with high confidence (0.93, 0.82, 0.94), demonstrating that the model handles visually distinct late-stage targets reliably. However, two partial diseased trees clipped by the image boundary in the upper-right and lower-right corners generate spurious low-confidence PWD-D detections. Because UAV imagery routinely contains partial canopies along tile edges, this failure mode has direct deployment implications and motivates either edge-aware post-processing or overlapping tiling at inference time.

Together, these three failure cases identify three remaining limitations: stage separation under canopy overlap, the inherent visual ambiguity of PWD-E targets at the few-pixel scale, and edge artifacts from partial canopies. None of the three is fully resolved by the current architecture, and each suggests a distinct direction for future work—instance-aware separation for overlapping crowns, multispectral or temporal cues to disambiguate genuine early chlorosis from natural canopy variation, and tile-aware inference strategies for production UAV pipelines.

From a robustness perspective, the six selected scenarios exhibit significant differences in terms of target size, background complexity, and target density. MSDF-Net maintains stable detection performance under these diverse conditions, demonstrating its good adaptability to different forest environments. Each category’s mAP in **Table 8** provides data support for this: the greatest improvement is seen in the PWD-E category, which directly aligns with the visual box diagrams shown in each Figure (A1), (B1), and (C2).

Across disease stages, background conditions, and target densities, the proposed modules consistently improve localization accuracy, classification reliability, and robustness under complex real-world conditions. This method is plug-and-play and can be easily integrated into various YOLO pine wood nematode disease detection frameworks. With a parameter count of 2.67M and computational cost of 11.7 GFLOPs, MSDF-Net falls within the range of existing lightweight edge-oriented detectors, making it a natural candidate for UAV-based PWD detection pipelines.

## 4 Discussion

In response to the challenges such as small target size and strong background interference in the detection of drone pine wilt disease, this study focused on feature extraction and feature fusion, and proposed the MSDF-Net model, which effectively enhanced the detection ability for small targets and disease areas in complex backgrounds.

### 4.1 Design rationale and module contributions

For small and irregularly-shaped targets, we adopted two strategies to enhance the feature representation. We integrated the C2f_DCNv4 module into the backbone network to achieve adaptive spatial sampling, thereby better capturing the unique irregular geometric variations and blurred boundaries of the diseased areas. Additionally, we introduced the P2 high-resolution detection layer in the detection head. This layer utilizes shallow feature maps with richer spatial details, significantly reducing the missed detection of early- infected trees that occupy only a few pixels. Although the P2 layer incurs some additional computational overhead, the improvement in recall rate and mAP indicates that the utilization of high-resolution features is crucial for addressing the challenges of small targets in this field.

To achieve robust feature aggregation in a complex environment, we introduced two components to suppress interference and maintain efficiency. The EMA attention mechanism is placed at the end of the feature extraction stage, used to recalibrate the feature responses in both spatial and channel dimensions, thereby enhancing the response to the affected areas and suppressing redundant background information. Meanwhile, we designed the C2f_DualConv module, which adopts a dual-branch convolution structure, capable of simultaneously capturing local spatial details and global context information, and reducing redundant computations. These two components work together to ensure the detection quality while meeting the efficiency requirements desirable for resource-constrained UAV platforms.

A key contribution of this work is the demonstrated transferability of the proposed modules across different YOLO versions. Unlike most existing studies that tightly couple their improvements to a specific detection framework, the two core modules proposed in this study—DCNv4 deformable convolution and the P2 detection layer—are structurally independent of the backbone block design. As validated in Section 3.4, integrating these two modules into YOLOv11n, YOLOv12n, and YOLOv13n yields consistent improvements of 6.1, 4.3, and 5.1 percentage points in mAP@0.5, respectively. Notably, the improvement on YOLOv11n is even larger than the 5.1 percentage point gain achieved with the full module set on YOLOv8n. This cross-version consistency suggests that the proposed modules address fundamental limitations in small-object detection and deformable feature modeling, rather than exploiting version-specific characteristics, and provides practitioners with flexible plug-and-play enhancements applicable to both current and future YOLO-based frameworks.

Despite these improvements, several limitations should be acknowledged. First, although the dataset encompasses three provinces with different pine species and climatic conditions, further validation on datasets from more diverse geographical regions and ecological environments (e.g., other species such as *Pinus tabuliformis* or *Pinus koraiensis*) would strengthen the generalization claim. Second, the incorporation of the P2 detection layer increases the computational cost, which may pose challenges for implementation on edge platforms with limited resources. In addition, all experiments in this study were conducted on a desktop-class GPU (NVIDIA GeForce RTX 4070 SUPER). Inference latency, throughput, memory footprint, and energy or power consumption on representative UAV-oriented edge platforms (e.g., NVIDIA Jetson Orin NX, Jetson Xavier NX) have not been benchmarked. The current characterization of MSDF-Net as “lightweight” is therefore based on indirect indicators—parameter count (2.67M) and FLOPs (11.7G)—which, while informative, do not substitute for on-device profiling. Furthermore, while the proposed DCNv4 and P2 modules have been validated across four YOLO versions (v8, v11, v12, v13), the present study does not evaluate transferability to architecturally distinct detection paradigms such as two-stage detectors (e.g., Faster R-CNN) or transformer-based detectors (e.g., DETR, RT-DETR). The cross-version generalization claim is therefore scoped to the YOLO family of single-stage anchor-free detectors, and broader cross-paradigm validation remains an open question. Finally, the current study focuses on single-disease detection and does not consider more complex scenarios such as multi-disease coexistence or long-term temporal monitoring.

The future work will be carried out along four directions. Firstly, we will combine cross-domain transfer learning and domain adaptation techniques to reduce the cost of re-labeling, and systematically evaluate and enhance the generalization ability of the model in unfamiliar ecological scenarios. Secondly, we will conduct on-device benchmarking on representative edge platforms (e.g., NVIDIA Jetson Orin NX) to measure inference latency, throughput, memory consumption, and energy or power profile. We will further explore compression methods such as pruning, TensorRT optimization, and quantization-aware training to reduce inference overhead while maintaining detection accuracy, thus bridging the gap toward real-time UAV deployment. Thirdly, we plan to extend this framework to multi-class forestry disease detection tasks, and examine the effectiveness of the proposed modules in scenarios with concurrent multiple diseases. Finally, we plan to extend the cross-version transferability validation beyond the YOLO family by integrating the DCNv4 and P2 modules into architecturally distinct detection paradigms, such as transformer-based detectors (e.g., RT-DETR) and two-stage detectors (e.g., Faster R-CNN), to assess whether the observed gains generalize to fundamentally different detection architectures.

### 4.2 Comparison with existing PWD detection studies

Recent studies have shown that, under specific experimental conditions, UAV-image-based identification of pine wilt disease can achieve relatively high detection accuracy. For example, Su et al. (2024) proposed PWD-YOLOv8n, which integrates CBAM, CA, BiFPN, and C2f-Faster-EMA into YOLOv8n and reports an mAP@0.5 of 94.3%. However, when comparing reported numbers across studies, caution is required: existing PWD detection works differ substantially in their datasets, ecological conditions, and task definitions, and these differences directly affect the interpretability of the reported metrics.

In particular, PWD-YOLOv8n is designed as a single-class detector that produces a binary healthy/diseased prediction, and its reported mAP@0.5 is therefore a single-class metric that does not involve inter-class confusion. In contrast, MSDF-Net addresses a four-class fine-grained detection problem (PWD-E, PWD-M, PWD-L, PWD-D) corresponding to disease progression stages, and its mAP is averaged over four classes. The two metrics are therefore structurally different, and a direct numerical comparison lacks methodological justification. Beyond the task definition itself, the two datasets also differ markedly in geographical coverage, tree species composition, image resolution, and annotation granularity. The dataset of Su et al. (2024) was collected only within the warm-temperate monsoon climate zone of Shandong Province, with images cropped to a fixed resolution of 1280 × 1280 pixels and binary labels. Our dataset spans three provinces (Jiangsu, Anhui, Liaoning) across both subtropical and temperate zones, uses UAV images resized to 640 × 640 pixels, and provides stage-specific annotations for four disease severity levels. These differences are summarized in **Table 10**.

**Table 10:** Comparison of experimental setup between Su et al. (2024) and this study.

Attribute	Su et al. (2024)	Ours
Geographic coverage	Shandong (Tai’an, Qingdao)	Jiangsu, Anhui, Liaoning
Climate zones	Warm temperate monsoon	Subtropical + temperate
Pine species	*P. massoniana*, *P. thunbergii*, *P. densiflora*	*P. massoniana*, *P. thunbergii*
Disease stages	Single class (diseased/healthy)	Four classes (PWD-E/M/L/D)
Training input size	1280×1280 px (cropped)	640×640 px (resized)
Annotation granularity	Binary bounding boxes	Stage-specific bounding boxes
Reported mAP@0.5	94.3% (single-class)	80.1% (multi-class, ours)

Given these fundamental differences, a meaningful comparison can only be drawn under controlled conditions. We therefore re-implemented PWD-YOLOv8n following the framework described by Su et al. (2024)—including CBAM, CA, C2f-Faster-EMA, BiFPN, and Inner-SIoU—and trained it on our cross-regional dataset under the same experimental settings as MSDF-Net, with identical data splits, hyperparameters, input resolution (640 × 640), and number of training epochs (200). The results are reported in **Table 11**.

**Table 11:** Controlled comparison with PWD-YOLOv8n on our cross-regional dataset under identical experimental settings.

Model	P (%)	R (%)	mAP@0.5 (%)	PWD-E AP	PWD-M AP	PWD-L AP	PWD-D AP	Params (M)	FLOPs (G)
YOLOv8n	72.2	70.1	75.0	39.2	82.5	95.0	83.4	3.01	8.1
PWD-YOLOv8n*	72.3	69.2	73.8	37.0	81.7	95.8	81.0	2.80	8.2
MSDF-Net	73.2	74.6	80.1	59.4	81.8	93.9	85.3	2.67	11.7

*Our re-implementation of PWD-YOLOv8n following Su et al. (2024), trained on our cross-regional dataset with the same data split, hyperparameters, input resolution (640×640), and number of training epochs (200) as MSDF-Net.

Several observations emerge from this controlled comparison. First, PWD-YOLOv8n achieves an mAP@0.5 of 73.8% on our dataset, substantially lower than the 94.3% reported in its original study. This gap confirms that mAP values are highly dataset-dependent and are not directly transferable across studies.

The drop can be attributed to the greater complexity of our dataset, which spans three provinces with distinct ecological conditions, contains four fine-grained disease stages rather than a binary classification, and uses 640 × 640 resized images that preserve the original field-of-view context but present smaller effective target sizes compared with the cropped 1280 × 1280 images used by Su et al. (2024).

Second, MSDF-Net achieves a higher overall mAP@0.5 than PWD-YOLOv8n on the same dataset (80.1% vs. 73.8%), with the most pronounced advantage observed in early-stage detection. The PWD-E AP of MSDF-Net (59.4%) exceeds that of PWD-YOLOv8n (37.0%) by 22.4 percentage points. This is the most practically meaningful improvement, as early detection provides the narrow time window necessary for intervention before irreversible tree mortality occurs. PWD-YOLOv8n relies primarily on attention-based feature refinement through CBAM, CA, and EMA, whereas MSDF-Net additionally incorporates DCNv4 deformable convolution for adaptive modeling of irregular disease boundaries and a P2 high-resolution detection layer for enhanced small-target sensitivity—two design choices that directly address the fundamental challenges most critical to early-stage detection.

In summary, the controlled comparison demonstrates that (1) a direct cross-study comparison of reported metrics is invalid due to fundamental differences in task definition and dataset characteristics; (2) when evaluated under identical conditions, MSDF-Net achieves superior overall performance, with the largest gains concentrated in the most challenging early-stage category; and (3) the architectural innovations in MSDF-Net—particularly DCNv4 and P2—provide benefits beyond those offered by attention-based refinement alone, validating the design rationale behind our approach.

## 5 Conclusion

This study proposes a cross-version transferable object detection framework named MSDF-Net, aiming to address the challenges in detecting target sizes that are small and background interference is strong in the detection of pine wood nematode disease by drones. This model integrates the C2f_DCNv4 module, the EMA attention mechanism, the C2f_DualConv module, and the P2 high-resolution detection layer. While maintaining lightweight design, it effectively enhances the model’s ability to extract and detect features in tiny diseased areas.

The experimental results show that MSDF-Net achieved an accuracy rate of 73.2%, a recall rate of 74.6%, and an mAP@0.5 of 80.1% on the cross-regional dataset. Compared with the baseline model YOLOv8n, this method reduced the number of parameters from 3.01 million to 2.67 million while increasing the mAP@0.5 by 5.1 percentage points and the recall rate by 4.5 percentage points. The greatest improvement was seen in the early disease detection (PWD-E), with an average precision (AP) increase of 20.2 percentage points. This leap demonstrates the model’s stronger perception of small targets, and early detection is the prerequisite for timely intervention.

To further validate the generalizability of the proposed method, the two core block-agnostic modules—DCNv4 deformable convolution and the P2 detection layer—were integrated into YOLOv11n, YOLOv12n, and YOLOv13n, yielding consistent improvements of 6.1, 4.3, and 5.1 percentage points in mAP@0.5, respectively. The proposed method has led to performance improvements in various YOLO versions because it addresses the common issues in small target detection and has good cross-version compatibility.

The visual results further confirmed the aforementioned performance improvement. In various scenarios including different disease stages, canopy shading, human structural interference, and different target densities, the MSDF-Net was able to accurately and stably perform detection. Moreover, the model has a relatively small number of parameters (2.67M) and moderate computational complexity (11.7 GFLOPs), positioning it as a promising candidate for future edge deployment. On-device benchmarking and model compression are planned as the next steps toward practical UAV-based large-scale forest health monitoring. These results are promising, but validation on datasets from additional geographical regions and pine species would strengthen the generalization claim further. Although several limitations remain—including the lack of on-device benchmarking, the YOLO-restricted scope of the transferability validation, and the absence of multi-disease evaluation—all of which are addressed in the Discussion as future work, the present results establish MSDF-Net as a practical and generalizable solution for UAV-based pine wilt disease detection, with strong potential for real-world deployment in intelligent forestry monitoring.

## Data Availability

The raw data supporting the conclusions of this article will be made available by the authors, without undue reservation.
